# Metabolic mechanisms orchestrated by Sirtuin family to modulate inflammatory responses

**DOI:** 10.3389/fimmu.2024.1448535

**Published:** 2024-09-20

**Authors:** Xiaoqing Li, Yunjia Li, Quan Hao, Jing Jin, Yi Wang

**Affiliations:** ^1^ Department of Hepatobiliary Surgery, The First Affiliated Hospital of USTC, Division of Life Sciences and Medicine, University of Science and Technology of China, Hefei, Anhui, China; ^2^ Key Laboratory of Immune Response and Immunotherapy, University of Science and Technology of China, Hefei, Anhui, China; ^3^ China Spallation Neutron Source, Dongguan, Guangdong, China

**Keywords:** SIRTs, epigenetics, post-translational modifications, metabolism, inflammation

## Abstract

Maintaining metabolic homeostasis is crucial for cellular and organismal health throughout their lifespans. The intricate link between metabolism and inflammation through immunometabolism is pivotal in maintaining overall health and disease progression. The multifactorial nature of metabolic and inflammatory processes makes study of the relationship between them challenging. Homologs of *Saccharomyces cerevisiae* silent information regulator 2 protein, known as Sirtuins (SIRTs), have been demonstrated to promote longevity in various organisms. As nicotinamide adenine dinucleotide-dependent deacetylases, members of the Sirtuin family (SIRT1–7) regulate energy metabolism and inflammation. In this review, we provide an extensive analysis of SIRTs involved in regulating key metabolic pathways, including glucose, lipid, and amino acid metabolism. Furthermore, we systematically describe how the SIRTs influence inflammatory responses by modulating metabolic pathways, as well as inflammatory cells, mediators, and pathways. Current research findings on the preferential roles of different SIRTs in metabolic disorders and inflammation underscore the potential of SIRTs as viable pharmacological and therapeutic targets. Future research should focus on the development of promising compounds that target SIRTs, with the aim of enhancing their anti-inflammatory activity by influencing metabolic pathways within inflammatory cells.

## 1 Introduction

Metabolism is crucial to cell development and function, with vital roles in cell growth, differentiation, function, immune responses, and other key processes (1, 2). Cell metabolism influences the development, differentiation, and activation of immune cells. Glucose, lipid, and amino acid metabolism are interconnected and play vital roles in inflammatory processes and immune system function (3–5). Glucose is the main source of energy for immune cells, especially during cytokine production and cell proliferation (6). Changes in glucose metabolism directly affect immune cell function and inflammatory response. In activated macrophages, glucose is converted to lactic acid primarily through glycolysis, a process known as the Warburg effect (7). It is also associated with the activation of signaling pathways such as phosphoinositide 3-kinase (PI3K)/protein kinase B (AKT)/mammalian target of rapamycin (mTOR) and hypoxia-inducible factor 1 (HIF-1), which play central roles in cell proliferation (8). Lipid metabolism plays a key role in regulating the immune response (9, 10). Not only does it provide energy for immune cells, but it also regulates signal transduction and gene expression of immune cells by influencing the structure and function of cell membranes (6). Amino acids are essential for the biosynthesis of proteins and other biomolecules required for immune cell function (6). Among them, glutamine is one of the main energy sources of lymphocytes and macrophages, and is essential for the proliferation and activation of immune cells (11). Branched chain amino acids (BCAAs), including leucine, isoleucine and valine, also essential in immune cell metabolism by supporting glycolysis, mitochondrial metabolism, and adenosine triphosphate (ATP) production (12, 13). The interactions between metabolisms and the immune system are complex and bidirectional (14). Dysregulation in one area can lead to imbalances in others, contributing to chronic inflammatory diseases and metabolic disorders. Thus, immunometabolism, which studies the intrinsic metabolic pathways of immune cells affecting their fate and function, has potential as a therapeutic target (15).

Inflammation is an innate immune mechanism important in restoring cell homeostasis (16). Inflammatory responses comprise several aspects, including inflammatory cells, mediators, and pathways, where inflammatory cells include macrophages, neutrophils, lymphocytes, and dendritic cells (DC) (17, 18). Inflammation also involves various cytokines and signaling pathways, including the nuclear factor kappa-B (NF-κB) pathway (19, 20), activation of which affects the expression of pro-inflammatory genes, such as those encoding growth factors, chemokines, and cytokines (21). Another important feature of the inflammatory process is macrophage activation and infiltration into resident tissues, which mediates local inflammation and is a hallmark of metabolic syndrome (22). Hence, inhibiting inflammation may be beneficial for improving the pathophysiology of inflammation-related diseases (23).

The interaction between metabolic changes and inflammation is an area of growing research interest. Inflammation responses and metabolic regulation are highly integrated and functionally dependent on one another. For example, the early initiation phase of acute inflammation is anabolic and primarily requires glycolysis, with reduced mitochondrial glucose oxidation for energy, whereas the later adaptation phase is catabolic, primarily requiring fatty acid oxidation as an energy source (24). Inflammatory cells require sufficient energy to maintain cellular integrity, as well as basic metabolism (25). For example, inflammatory cells produce energy through glycolysis, to maintain their specific functions, such as migratory movement and activation. In addition, immune cells metabolically transform glucose, lipids, and proteins through the tricarboxylic acid (TCA) cycle. If glucose or lipid metabolism are abnormal, inflammatory cell metabolism can be blocked or continuously activated, leading to chronic inflammation. There is strong evidence that the metabolic state of inflammatory cells is critical to their function. For example, pro-inflammatory cells, such as M1 macrophages and activated DC, are more inclined to glycolytic activity, whereas anti-inflammatory cells, including regulatory T cells (Tregs) and M2 macrophages, tend toward oxidative phosphorylation, possibly related to the fact that metabolites of glycolysis and TCA cycle have different signaling effects (26). In summary, metabolism is intricately linked with inflammation and immune system. Disruptions in these metabolic pathways can lead to chronic diseases, highlighting the importance of managing inflammation and metabolism in clinical practice. For example, metformin, traditionally used for diabetes, has shown promise in treating autoimmune diseases by mediating metabolic pathways in T lymphocytes and innate immune cells (27).

*Saccharomyces cerevisiae* silent information regulator 2 (Sir2) protein is the founding Sirtuin family member and functions in transcriptional silencing via inducing formation of heterochromatin domains and as a pro-longevity factor for replicative lifespan in yeast (28). Human SIRTs, the closest homologs of the Sir2, remodel chromatin by deacetylating histones and suppresses transcription by forming heterochromatin (29). The SIRTs is a class of nicotinamide adenine dinucleotide (NAD^+^)-dependent deacetylases that regulate various metabolic pathways (30). SIRTs expression and activity are closely related to metabolic intermediates, such as NAD^+^ and acyl-CoA molecules. NAD^+^ is a cofactor in numerous enzymatic processes and regulates key metabolic pathways (31), where balance between its oxidized (NAD^+^) and reduced (NADH) forms is critical for proper cellular function and survival. Caloric restriction (CR) has the capacity to elevate intracellular levels of NAD^+^ by catalyzing nicotinamide phosphoribosyl transferase (NAMPT), the rate-limiting enzyme in NAD^+^ synthesis (32). Conversely, NAD^+^ is downregulated during aging, and associated with various markers of age-related metabolic decline, immunosenescence, and chronic inflammation (33, 34), where SIRTs activities are simultaneously decreased (35). Acyl-CoA molecules have significant roles in numerous physiological and metabolic processes, and their levels are crucial for evaluating metabolic health and controlling post-translational modifications (PTMs) (36, 37). SIRTs mediate the process of PTMs of proteins, which can regulate the expression of specific genes, including those encoding metabolic factors, thus determining cell fate via epigenetic mechanisms; for example, SIRTs mediate deacetylation, defatty-acylation, adenosine diphosphate (ADP)-ribosylation, desuccinylation, deglutarylation, and demalonylation, among other processes (38–40). More importantly, Hallows et al. found that mammalian SIRTs could directly control the activity of metabolic enzymes; for instance, SIRT1 deacetylates acetyl-CoA synthetases 1(AceCS1) on K661 in cytoplasm and SIRT3 deacetylates AceCS2 on K635 in mitochondrial (41). Hence, SIRTs determine cell fate by directly regulating protein PTMs and indirectly regulating cell energy metabolism by using intracellular NAD^+^ to produce *O*-acetyl-ADP-ribose.

The SIRTs is closely associated with aging, which is also linked to chronic, low-grade inflammation (42, 43). SIRTs are involved in inflammation via various mechanisms, such as NF-κB and NOD-like receptor thermal protein domain associated protein 3 (NLRP3) pathways (30). SIRTs are dispersed throughout the subcellular compartments of cells. SIRT1, SIRT6, and SIRT7 are normally found in the nucleus; SIRT6 is distributed throughout the nucleus, while SIRT7 is concentrated in the nucleolus. SIRTs localized to cell nuclei can participate in modulating inflammatory gene expression to regulate the development of inflammation. SIRT2 resides in the cytoplasm, whereas SIRT3, SIRT4, and SIRT5 localize to the mitochondria. Moreover, SIRTs exhibit differential expression depending on various cell types and tissues [e.g., B lymphocytes and breast cancer (BC) tissues], impacting cellular processes and the cell fate (44, 45). The diverse distribution and expression of SIRTs allows them to contribute to various cellular functions, including energy metabolism and inflammation. In addition to aging and inflammation, SIRTs have regulatory roles in the occurrence and development of diverse diseases, including tumors, cardiovascular diseases, and respiratory conditions, among others, and are thus considered therapeutic targets (30). Therefore, the SIRTs are highly anticipated as potential therapeutic targets and SIRTs modulators are undergoing development with the aim of clinical application.

SIRTs can mediate the development of inflammation by regulating metabolism (e.g., senescence, CR, oxidative stress, and Warburg effect). Senescence promotes metabolic disease, which in turn promotes senescence (35). With advancing age, intracellular macromolecules undergo damage, while physiological repair and autophagic capacities diminish. Accumulated macromolecular damage triggers innate immune system activation, leading to inflammation (46, 47). Hence, elimination of inflammation is a potential anti-aging strategy (47). The altered glycolysis and fatty acid oxidation observed in acute/chronic inflammation are, at least partially, attributable to SIRTs (48). CR can modulate immune cell types and proportions (49–51), alter the composition of the gut flora and also affect the systemic immune system (49). SIRT1 and SIRT3 have central roles in CR. For instant, SIRT1 enhances skeletal muscle insulin sensitivity in mice during CR (52). Additionally, SIRT3 mediates reduction of oxidative damage and prevention of age-related hearing loss under CR (53). Moreover, oxidative stress can cause production of endogenous damage associated molecular patterns (DAMPs) and cytokine release, causing metabolic imbalance and inflammatory aging (47). SIRTs play crucial roles in modulating oxidative stress and inflammatory responses. For instance, SIRT2, SIRT3, SIRT5, and SIRT6 participate in inflammatory and immune reactions by mediating oxidative stress. Lastly, the interplay between SIRTs and the Warburg effect has emerged as a critical regulatory mechanism in metabolism and inflammation, which is a phenomenon that is pivotal in the pathophysiology of various diseases. Therefore, SIRTs are promising targets in metabolic-mediated inflammation. Some crucial small molecules involved in metabolic pathways affect the epigenetic regulation function of SIRTs and determine cell fate by regulating PTMs of proteins and the transcription of various genes. Despite these advances, there are still many issues to be elucidated.

In this review, we discuss the roles of the SIRTs in metabolism and elaborate on SIRTs participation in inflammatory responses from four perspectives: metabolism, inflammatory cells, mediators, and signaling pathways. By focusing on the associations between inflammation and metabolic pathways, we aim to explore how the SIRTs regulate cell metabolism by functioning as epigenetic erasers, thus influencing the occurrence and development of inflammation, and delaying senescence.

## 2 Nuclear SIRT1, SIRT6, and SIRT7 frequently regulate glucose and lipid metabolism

### 2.1 SIRT1

**Figure 1:**
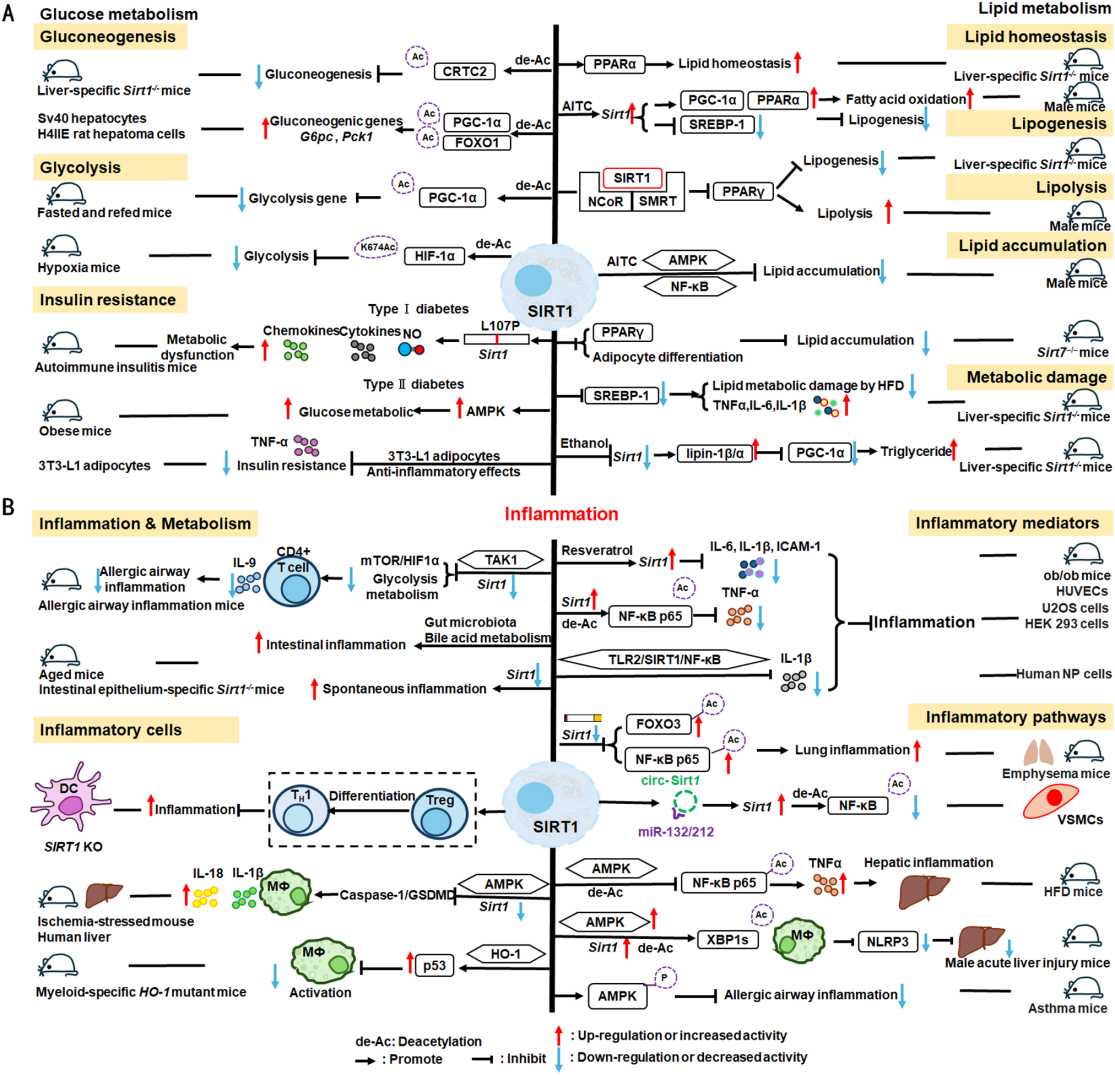
Overview of the roles SIRT1 in metabolism and inflammation. **(A)** Effects of SIRT1 in glucose metabolism, including gluconeogenesis, glycolysis, and insulin resistance and SIRT1-regulated lipid metabolism, including lipid homeostasis, lipogenesis, lipolysis, and lipid accumulation. **(B)** Effects of SIRT1 in regulating inflammation. SIRT1 has important roles in inflammation via the glycolysis, bile acid metabolism, NF-κB, and AMPK pathways. SIRT1 also participates in inflammatory signaling regulation in macrophages and DC, and in modulating IL-6, ICAM-1, TNF-α, and IL-1β.

Nuclear SIRT1 exerts considerable influence on metabolism and inflammation, as illustrated in **Figure 1**. SIRT1 regulates energy homeostasis in various metabolic tissues by modulating several signaling pathways, such as those involved in glucose and lipid metabolism. The effects of SIRT1 on glucose metabolism have been identified through its deacetylation, including up- or down-regulation of gluconeogenesis, glycolysis, and insulin homeostasis. Regulation of lipid metabolism by SIRT1 includes maintaining lipid homeostasis, suppressing lipogenesis and lipid accumulation, as well as facilitating lipolysis. SIRT1 also plays an important role in inflammation via glycolysis, bile acid metabolism, NF-κB, and AMP-activated protein kinase (AMPK) pathways. SIRT1 also participates in inflammatory signaling regulation in macrophages and DC as well as modulating the secretion of inflammatory cytokines.

#### 2.1.1 Regulation of glucose metabolism

Gluconeogenesis, glycolysis, and insulin resistance are major aspects of glucose metabolism involving SIRT1. SIRT1 has a dual role in gluconeogenesis (54), with SIRT1-mediated deacetylation of cAMP-responsive element binding (CREB)-regulated transcription coactivator 2 (CRTC2) reducing the effect of glucagon to negatively regulate gluconeogenesis in liver-specific *Sirt1* ^-/-^ mice (55). However, SIRT1 enhances transcription of the gluconeogenic genes, *G6pc* and *Pck1*, by deacetylating peroxisome proliferator-activated receptor γ coactivator-1α (PGC-1α) and Forkhead box O1 (FOXO1) in Sv40 hepatocytes and H4IIE rat hepatoma cells (56, 57). SIRT1 can act as a cofactor for PGC-1α both positively and negatively in the control of gene expression (57). SIRT1 also modulates glycolysis, which is a key component of the Warburg effect (58); for example, SIRT1 regulates the acetylation level of PGC-1α to inhibit glycolysis-associated genes during fasting (57). Furthermore, HIF-1α, a master regulator of glycolytic genes, is deacetylated by SIRT1 at lysine 674 (K674) to repress HIF-1α target genes and suppress glycolysis (59). However, during hypoxia, NAD^+^ level decreases and the activity is inhibited, thereby stimulating HIF-1α-mediated anaerobic glucose metabolism (59). Moreover, impaired glucose metabolism and insulin resistance can cause metabolic diseases, such as diabetes. SIRT1 has an important role in type I diabetes. The single amino acid substitution, SIRT1-L107P, enhances hyperinflammation and metabolic dysfunction by inducing production of nitric oxide, cytokines, and chemokines in autoimmune insulitis mice (60). Additionally, SIRT1 regulates glucose metabolism by upregulating AMPK pathway, which can improve the glucose-related metabolic imbalance in type II diabetes in obese mice (30, 61). SIRT1 also alleviates tumor necrosis factor-α (TNF-α)-induced insulin resistance partly through the anti-inflammatory effects of 3T3-L1 adipocytes (62). Therefore, appropriate and sufficient SIRT1 expression is conducive to maintenance of blood glucose stability by regulating insulin signaling proteins. Hence, development of SIRT1 activators as potential treatments for diabetes warrants exploration in the future (63).

#### 2.1.2 Regulation of lipid metabolism

The liver is among the major organs in which SIRT1 plays an essential role regulating lipid metabolism via modifying the acetylation status of a wide range of molecules (64–66). For example, SIRT1 regulates lipid homeostasis by regulating peroxisome proliferators-activated receptor α (PPARα) and fatty acid metabolism (65). SIRT1 inhibits peroxisome proliferators-activated receptor γ (PPARγ) by binding to nuclear receptor co-inhibitor (NCoR) and silencing mediator of retinoid and thyroid hormone receptors (SMRT), thereby reducing lipogenesis and enhancing lipolysis, which is associated with increased lifespan in mammals (67). In *Sirt7* ^−/−^ mice, SIRT1 blocks PPAR-γ and adipocyte differentiation, thereby diminishing white fat accumulation (68). SIRT1 ameliorates liver metabolic damage caused by high-fat-diet (HFD) by decreasing sterol regulatory element-binding protein 1 (SREBP1) expression and improving antioxidant and anti-inflammatory status (66). In mice, *Sirt1* deletion disrupts lipin-1β/α signaling, increases inflammatory gene expression levels, and aggravates alcoholic fatty liver (64). Moreover, ethanol exposure disrupts SIRT1 activity and contributes to alcoholic liver disease in rodents. In nonalcoholic fatty liver disease, allyl isothiocyanate (AITC) ameliorates lipid accumulation and inflammation through the SIRT1/AMPK and NF-κB signaling pathways (69).

Therefore, SIRT1 activity is closely related to regulation of glucose metabolism and lipid metabolism in metabolic diseases, such as diabetes and liver disease. Simultaneously, in inflammatory responses, cells respond in different ways to external stimuli through various SIRT1-regulated metabolic pathways. Specific details are discussed below.

#### 2.1.3 SIRT1 mediated inflammation regulation

SIRT1 regulates inflammatory processes through metabolic pathways, such as glycolysis and bile acid metabolism. During Th9 cell differentiation, SIRT1 is selectively suppressed by transforming growth factor-β (TGF-β)-activated kinase 1 (TAK1), which negatively regulates mTOR and HIF-1α signaling activity, together with glycolysis metabolism, suppresses CD4^+^ T cell interleukin 9 (IL-9) production, alleviates allergic airway inflammation, and promotes malignant tumor growth (70). Bile acids are crucial for intestinal nutrient absorption and biliary cholesterol secretion, and changes in bile acids are associated with metabolic disease (71). Nie et al. found that gut symbionts can remodel intestinal flora and reduce chronic inflammation through a secondary bile acid (3-sucCA) biosynthetic pathway, thereby reversing metabolic dysfunction-associated steatohepatitis development (72). Additionally, SIRT1 is a mediator of host-microbiome interactions, and involved in intestinal inflammation regulation through modulation of gut microbiota and bile acid metabolism via the hepatocyte nuclear factor 1α (HNF1α) pathway in mice (73). Deletion of *Sirt1* in aged mice activates intestinal secretory cells and induces spontaneous inflammation (73). These observations cast light on the role of metabolism during inflammation.

SIRT1 has critical roles in inflammatory processes, by influencing inflammatory cells, mediators, and pathways. For example, DC SIRT1 has a central role in regulating T cell differentiation to modulate inflammatory signaling (30, 74). *SIRT1* knockout (KO) in DC restrained generation of anti-inflammatory forkhead box protein P3 (Foxp3)-positive Tregs while driving proinflammatory T helper type 1 cell development, which enhanced T-cell-mediated anti-microbial inflammatory responses (74). In addition to DC, SIRT1 also influences macrophage function through the NF-κB, AMPK, and p53 pathways, as elaborated in detail below.

SIRT1 also has vital roles in inflammation via mediators, such as TNF-α, IL-1β, and leukocyte/platelet adhesion and E-selectin/intercellular adhesion molecule (ICAM-1), among other factors. SIRT1 activation enhances deacetylation of the p65 protein, inhibiting TNF-α-induced NF-κB transcriptional activation, and reducing the secretion of lipopolysaccharide (LPS)-stimulated TNF-α (75, 76). Further, SIRT1 exerts anti-inflammatory effects against IL-1β-mediated pro-inflammatory stress through the Toll-like receptor 2 (TLR2)/SIRT1/NF-κB pathway in human degenerative nucleus pulposus (NP) cells (77). *Sirt1* deficiency increases microvascular inflammation in obese mice with sepsis; however, SIRT1 expression is increased by resveratrol, and that of ICAM-1 is downregulated under resveratrol treatment in ob/ob mice (78). Furthermore, SIRT1 inhibits inflammation by decreasing pro-inflammatory cytokines, such as IL-6, IL-1β, and ICAM-1 in human umbilical vein endothelial cells (HUVECs) (79).

SIRT1 has an established role in anti-inflammatory effects via the NF-κB, AMPK, p53, and NLRP3 pathways. SIRT1 can suppress inflammation in multiple tissues and macrophages by interacting with NF-κB p65 and deacetylating RelA/p65 to inhibit NF-κB-associated transcription (77, 80). Under cigarette smoke or oxidative/carbonyl stress, SIRT1 is reduced and FOXO3 and NF-κB acetylation is upregulated, leading to premature lung aging and inflammation (81). Furthermore, in atherosclerosis, circ-SIRT1, derived from the *SIRT1* gene, contributes to inhibiting NF-κB activation by direct interaction and promoting SIRT1 expression through competitively binding to miR-132/212 in vascular smooth muscle cells (82); however, AMPK signaling negatively regulates the NF-κB-TNFα inflammatory axis through increasing SIRT1 deacetylase activity and inhibiting NF-κB transcription activity by deacetylating p65, facilitating TNF-α signaling and triggering hepatic inflammation and fibrosis development (83). Moreover, SIRT1 enhances AMPK phosphorylation to weaken the oxidative stress response, and inhibit allergic airway responses and inflammation (84). In the acute liver ischemia/reperfusion (I/R) injury phase, Ikaros signaling favors sterile inflammation in conjunction with negative regulation of SIRT1 via AMPK, resulting in canonical inflammasome-pyroptosis pathway activation, leading to release of IL-1β and IL-18 (85). Besides, heme oxygenase-1 (HO-1) positively regulates SIRT1, while SIRT1-induced ADP-ribosylation factor (Arf) inhibits activity of the E3 ubiquitin-protein ligase, MDM2, to stabilize p53, thereby attenuating macrophage activation in myeloid-specific *HO-1* mutant mice (86). Finally, SIRT1 is reported to play important roles in the anti-inflammation effects mediated by attenuation of activity of NLRP3, the best-characterized inflammasome (87). SIRT1 activation and multipotent mesenchymal stromal/stem cell-mediated AMPK activity in macrophages results in spliced X-box binding protein 1 (XBP1s) deacetylation and subsequent NLRP3 inflammasome inhibition in acute liver injury mice (88).

In summary, SIRT1 regulates inflammation through multiple routes. First, SIRT1 can modulate metabolic pathways, such as mTOR and HIF-1α signaling pathway, glycolysis and bile acid metabolism, to alleviate allergic airway and intestinal inflammation. Second, SIRT1 can reduce macrophage activation by stabilizing the p53 pathway and modulating T cell-associated inflammation in DC. SIRT1 also plays a vital role in inflammation via inflammatory mediators, such as TNF-α, IL-1β, and ICAM-1, among others. SIRT1 primarily regulates inflammation through the NF-κB, AMPK, p53, and NLRP3 pathways. For example, SIRT1 inhibits NF-κB-mediated inflammatory cytokine expression by downregulating p65 acetylation through its deacetylation at K310 (89). Moreover, AMPK is an important NF-κB inhibitor, and SIRT1 indirectly regulates NF-κB signaling and inhibits inflammation by activating AMPK (90). Further, SIRT1 regulates nonalcoholic fatty liver disease development via the NF-κB and AMPK pathways. Overall, the evidence points to a role for SIRT1 as a bridge between metabolism and inflammation, making it a potential therapeutic target in metabolic and immune diseases; however, the specific role and mechanisms involving SIRT1 in this promising field require further study.

### 2.2 SIRT6

**Figure 2:**
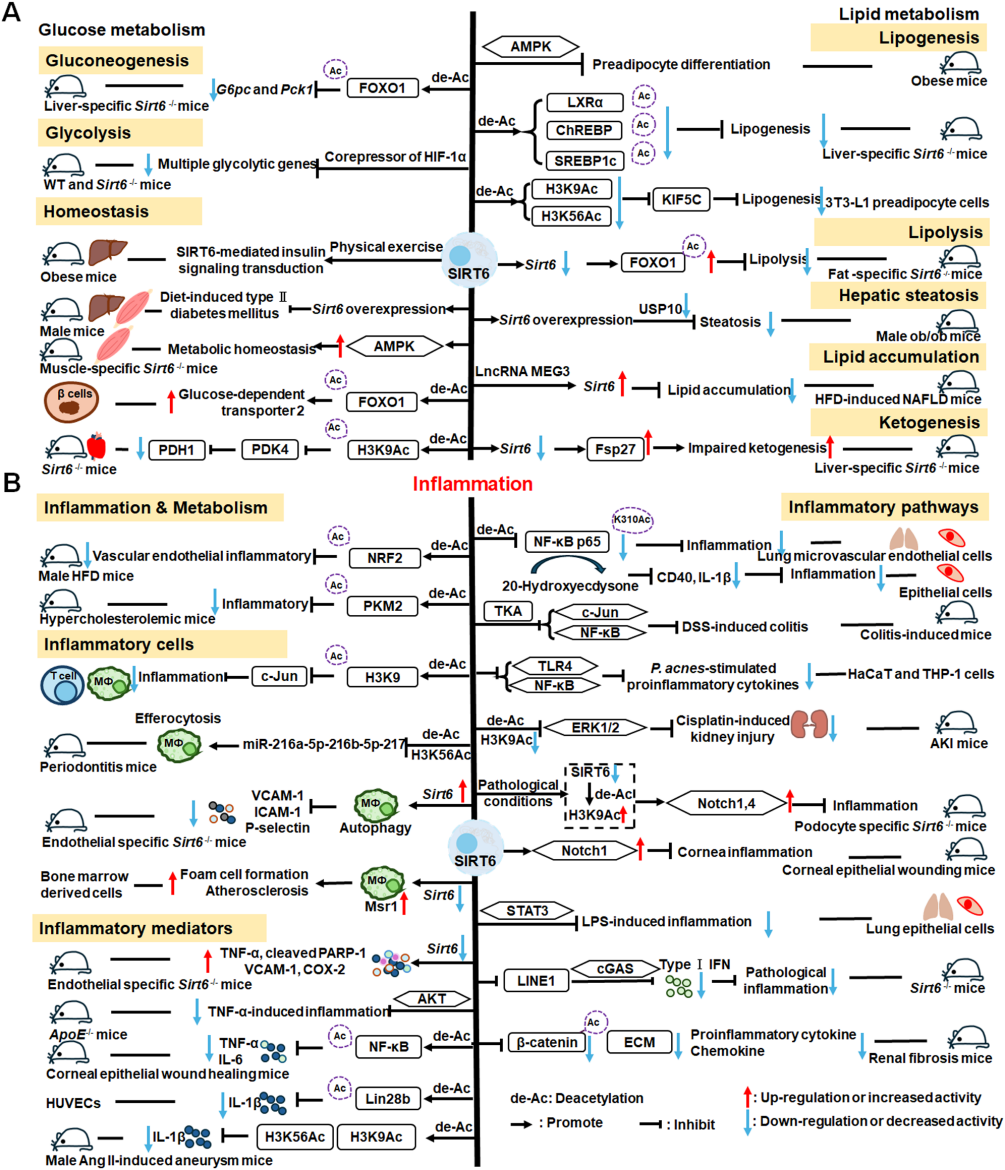
Overview of the roles of SIRT6 in metabolism and inflammation. **(A)** SIRT6 in glucose and lipid metabolism. Regulation of glucose metabolism by SIRT6, including gluconeogenesis, glycolysis, and metabolism homeostasis is illustrated. The figure also shows the regulatory effects of SIRT6 in lipid metabolism, including lipogenesis, lipolysis, steatosis, lipid accumulation, and ketogenesis. **(B)** SIRT6 in regulating inflammation. SIRT6 participates in anti-inflammation mechanisms via metabolic pathways, such as NRF2 and PKM2 signaling. Moreover, SIRT6 is involved in regulation of inflammatory responses mediated by immune cells, including T cells and macrophages. SIRT6 also inhibits inflammation by decreasing pro-inflammatory cytokines, such as TNF-α, IL-6, IL-1β, and ICAM-1. Finally, SIRT6 modulates inflammation via various signaling pathways.

SIRT6 has established roles in metabolism and inflammation, as illustrated in **Figure 2** (91). There is increasing evidence that SIRT6 has crucial functions in glucose and lipid metabolism (92). The regulation of glucose metabolism by SIRT6 includes inhibition of gluconeogenesis and glycolysis, as well as the maintenance of metabolic homeostasis. SIRT6 regulates lipid metabolism by exerting its deacetylation activity, including lipogenesis, lipolysis, and lipid accumulation, as well as lipid homeostasis, and ketogenesis. At the same time, SIRT6 participates in anti-inflammatory processes via metabolic pathways such as the nuclear factor erythroid 2-related factor (NRF2) and pyruvate kinase isozyme type M2 (PKM2) signaling pathways. Furthermore, SIRT6 plays a crucial role in regulating inflammatory responses mediated by immune cells. Moreover, SIRT6 reduces pro-inflammatory cytokines expression mainly through its deacetylation and impacts various signaling pathways to inhibit inflammation, such as NF-κB, extracellular regulated protein kinases (ERK)1/2, Notch, signal transducer and activator of transcription (STAT3), and cyclic GMP-AMP synthase (cGAS) pathways, among others.

#### 2.2.1 Regulation of glucose metabolism

In this paper, we discuss the involvement of SIRT6 in three aspects of glucose metabolism: gluconeogenesis, glycolysis, and metabolic homeostasis. In gluconeogenesis, unlike SIRT1, SIRT6 down-regulates *G6pc* and *Pck1* transcription through deacetylation of FOXO1 (93), while in glycolysis, SIRT6 acts as a corepressor of the master glycolysis regulator, HIF-1α, and inhibits the expression of multiple glycolytic genes (94). SIRT6 also contributes to maintenance of glucose metabolism homeostasis in the whole body, liver, skeletal muscle, pancreas, heart, and other tissues (95, 96). Zhang and colleagues reported that physical exercise intervention ameliorates insulin resistance and improves SIRT6-mediated insulin signaling transduction in the liver of obese rats (92). Moderate SIRT6 overexpression can increase insulin sensitivity in skeletal muscle and liver, which has a protective effect against diet-induced type II diabetes mellitus (97). Further, SIRT6 regulates AMPK activation to modulate metabolic homeostasis in skeletal muscle (96). Besides, SIRT6 deacetylates FOXO1 and then increases glucose-dependent transporter 2 expression to maintain glucose sensing and systemic glucose tolerance in pancreatic β cells (98). In the heart, SIRT6 deacetylates H3K9Ac in the pyruvate dehydrogenase kinase 4 (*PDK4*) promoter to downregulate PDK4 levels, thus decreasing pyruvate dehydrogenase 1 (PDH1) activity and ATP production by regulating mitochondrial oxidative phosphorylation (95). In addition, some compounds affect glucose metabolism homeostasis by targeting SIRT6. For example, the SIRT6−specific inhibitor, OSS−128167, exacerbates diabetic cardiomyopathy in a mouse model of streptozotocin−induced diabetes and high glucose−treated cardiomyocytes (99). Additionally, cyclosporine A suppresses *SIRT6* expression and subsequently fuels glycolysis and the TCA cycle, as well as HIF-1α expression, in neutrophils (100). This finding suggests that SIRT6 can regulate metabolism in inflammatory cells, such as neutrophils, to maintain metabolic homeostasis. In the future, we hope that the regulation of SIRT6 in glucose metabolism will be extended to all kinds of immune cells.

#### 2.2.2 Regulation of lipid metabolism

SIRT6 has been implicated in the regulation of lipid metabolic processes, including lipogenesis, lipolysis, hepatic steatosis, lipid accumulation, and ketogenesis (101). Hong et al. found that SIRT6 inhibits preadipocyte differentiation by activating the AMPK pathway (102). To inhibit hepatic lipogenesis, SIRT6 suppresses the transcriptional activities of liver X receptor α (LXRα), carbohydrate response element binding protein (ChREBP), and SREBP1 through direct deacetylation (101). Moreover, Chen et al. discovered that SIRT6 negatively regulates a repressor of mitotic clonal expansion important for lipogenesis, kinesin family member 5C (KIF5C), through deacetylation of H3K9 and H3K56 (103). Further, mice with fat-specific *Sirt6* knockout (FKO) exhibit increased FOXO1 acetylation and reduced adipose triglyceride lipase (ATGL) expression (104). FKO also increases inflammation in adipose tissue, leading to insulin resistance by inhibiting lipolysis. Furthermore, ubiquitin-specific peptidase 10 (USP10) interacts with SIRT6 and inhibits its ubiquitination and degradation (104), while Sirt6 overexpression attenuates USP10 deficiency-potentiated hepatic insulin resistance, steatosis, and inflammation (105). In terms of lipid accumulation, long non-coding RNA maternally expressed gene 3 (LncRNA MEG3) up-regulates *Sirt6* by ubiquitinating enhancer of zeste homolog 2 (EZH2), which suppresses lipid accumulation and inflammation *in vitro* and alleviates nonalcoholic fatty liver disease *in vivo (* 106). Besides, mice with hepatocyte-specific *Sirt6* deficiency exhibit impaired ketogenesis, by enhancing expression of fat-specific induction of protein 27 (Fsp27), which regulates lipid metabolism (107).

Hence, SIRT6 is involved in glucose and lipid metabolism via both direct and indirect means, and is expected to become a therapeutic target in the treatment of metabolic diseases in the future. Unraveling how SIRT6 regulates inflammation through metabolic pathways is a likely future research hotspot.

#### 2.2.3 SIRT6 mediated inflammation regulation

Metabolic regulation and inflammatory responses are highly integrated and functionally interdependent. NRF2 and PKM2 are two notable examples at the intersection between metabolic regulation and inflammation. NRF2 is a key transcription factor that regulates antioxidant stress, with significant roles in antioxidant and anti-inflammatory responses (108, 109). Microglia SIRT6 maintains its stability by deacetylating NRF2, thereby achieving anti-oxidation and anti-inflammation functions and ultimately regulating the damage caused by HFD-induced obesity (110). Through positively regulating NRF2 phosphorylation and stimulating transcription of its target genes, SIRT6 can attenuate the vascular endothelial inflammatory response (111, 112). In addition, certain compounds and medicines contribute to modulating the NRF2 pathway. Orientin, a natural flavonoid compound, attenuates IL-1β-induced inflammation and degradation of the extracellular matrix by activating the NRF2/HO-1 and SIRT6 signaling pathways (113). Besides, *Gami-Yukmijihwang-Tang*, a Korean medicine, can protect against LPS-induced neuroinflammation and oxidative stress by regulating the *Sirt6*-related NRF2/HO-1 signaling pathway and normalizing the glutathione (GSH) redox cycle (114). PKM2 is a glycolytic enzyme that is also a potential target for regulating inflammatory responses. SIRT6 can deacetylate PKM2, while hydroxytyrosol acetate exerts an inhibitory effect on the inflammatory response in TNF-stimulated HUVECs and the thoracic aorta of hypercholesterolemic mice, partly through SIRT6-mediated PKM2 signaling pathway (91).

SIRT6 is also involved in regulating inflammatory responses in inflammatory cells. For example, SIRT6 suppresses c-Jun-dependent expression of proinflammatory genes in T cells and macrophages and inhibits chronic inflammation and fibrosis in the liver (115). SIRT6 epigenetically regulates macrophage efferocytosis and controls inflammation resolution in diabetic periodontitis by inhibiting transcription of the miR-216a-5p-216b-5p-217 cluster through acetylated H3K56 (116). Besides, SIRT6 plays a critical role in cardiovascular diseases by regulating the inflammatory response in macrophages and vascular endothelial cells. Sirt6 overexpression in atherosclerosis promotes macrophage autophagy and inhibits the expression of vascular cell adhesion molecule-1 (VCAM-1), ICAM-1, and platelet selectin (P-selectin), leading to reduced macrophage infiltration of macrophages and plaque stabilization (117). Loss of SIRT6 in bone marrow-derived cells stimulates c-MYC transcription, thereby enhancing macrophage scavenger receptor 1 (Msr1) levels and increasing oxidized low-density lipoprotein to enhance foam cell formation and atherosclerosis (118).

In addition, SIRT6 inhibits inflammation by decreasing levels of pro-inflammatory cytokines, such as TNF-α, IL-6, IL-1β, and ICAM-1 (79). In mice, lack of endothelial *Sirt6* increases tissue factor and NF-κB-associated pro-inflammatory cytokines, including TNF-α, cleaved poly ADP-ribose polymerase (PARP-1), VCAM-1, and the COX-prostaglandin system (COX-2), thereby accelerating thrombosis via induction of the coagulation cascade (119). SIRT6 also inhibits TNF-α-induced inflammation of vascular adventitial fibroblasts through reactive oxygen species (ROS) and AKT signaling pathway (120). SIRT6 suppresses inflammatory responses and downregulates expression of the inflammatory factors, TNF-α and IL-6, via the NF-κB pathway (121). Additionally, Yao et al. found that SIRT6, which can deacetylate Lin28b, inhibits vascular endothelial cell pyroptosis by decreased numbers of propidium iodide-positive cells and reduced gastrin D (GSDMD) cleavage, as well as downregulated lactate dehydrogenase and IL-1β release in TNF-α-induced vascular endothelial cells (122). SIRT6 can bind to the IL-1β promoter and repress IL-1β expression, partly by reducing H3K9 and H3K56 acetylation and inhibiting inflammation and senescence in thoracic aortic aneurysms (123).

Recently, extensive studies have clearly shown that SIRT6 mainly regulates anti-inflammatory effects via inhibiting various pathways, including the NF-κB, ERK1/2, Notch, STAT3, and cGAS pathways, among others. SIRT6 protects against LPS-induced lung microvascular endothelial cell inflammation via suppressing NF-κB activation (124), while 20-Hydroxyecdysone inhibits inflammation in endothelial cells through SIRT6-mediated deacetylation of NF-κB p65 K310, to reduce CD40 expression and IL-1β level (125). *Sirt6* overexpression attenuates dextran sulfate sodium (DSS)-induced colitis by regulating TAK1 to inhibit NF-κB and c-Jun signaling in mice (126). *SIRT6* overexpression also attenuates *Propionibacterium acnes*-stimulated proinflammatory cytokine production and reduces inflammation-associated NF-κB activation, the TLR4 signaling, and ROS signaling in HaCaT and THP-1 cells (127). SIRT6 can activate autophagy and inhibit the ERK1/2 pathway by negatively regulating H3K9Ac levels in the ERK1/2 promoter, thereby attenuating cisplatin-induced kidney injury, in terms of renal dysfunction, inflammation, and apoptosis (128); however, in LPS-induced acute respiratory distress syndrome, SIRT6 expression is significantly down-regulated, thereby activating the ERK1/2 pathway and exacerbating inflammation, apoptosis, and tight junction protein loss (129). Under normal conditions, SIRT6 inhibits Notch1 and Notch4 transcription by decreasing H3K9Ac levels, while under pathological conditions, reduced SIRT6 enhances Notch1 and Notch4 transcription and induces inflammation, apoptosis, and actin cytoskeleton disruption, as well as inhibiting autophagy-mediated podocyte injury (130). SIRT6 is also an important regulator of inflammation in the cornea, where Sirt6 deletion impairs corneal epithelial wound healing by upregulating inflammatory responses and reducing Notch1 signaling (131). Besides, SIRT6 attenuates LPS-induced inflammation and lung epithelial cell apoptosis in acute lung injury (ALI) by inhibiting angiotensin converting enzyme 2 (ACE2)/STAT3/Pim-1 proto-oncogene, serine/threonine kinase (PIM1) signaling (132). *Sirt6^-/-^* mice accumulate long interspersed nuclear element 1 (LINE1) cDNA, triggering type I interferon (IFN) response via the cGAS DNA sensing pathway, resulting in pathological inflammation (133). Loss of proximal tubular SIRT6 aggravates unilateral ureteral obstruction-induced tubulointerstitial inflammation and fibrosis by increasing β-catenin acetylation, extracellular matrix protein expression, proinflammatory cytokines, and chemokine expression (134).

Overall, SIRT6 affects the occurrence and development of inflammation through metabolic pathways, such as NRF2 and PKM2 signaling. In addition, SIRT6 maintains appropriate inflammatory responses by regulating various inflammatory cells, factors, and signaling pathways. Specifically, SIRT6 can mediate the secretion of inflammatory factors by regulating macrophage efferocytosis and autophagy or acting on the promoter of inflammatory factors. Mechanistically, SIRT6 blocks the NF-κB, TLR4, ERK1/2, STAT3, and cGAS signaling pathways in various disease models. Future research should focus on whether known SIRT6 activators influence anti-inflammatory activity by contributing to metabolic pathways involving inflammatory cells, which represent common and promising targets in metabolic diseases and inflammatory responses.

### 2.3 SIRT7

**Figure 3:**
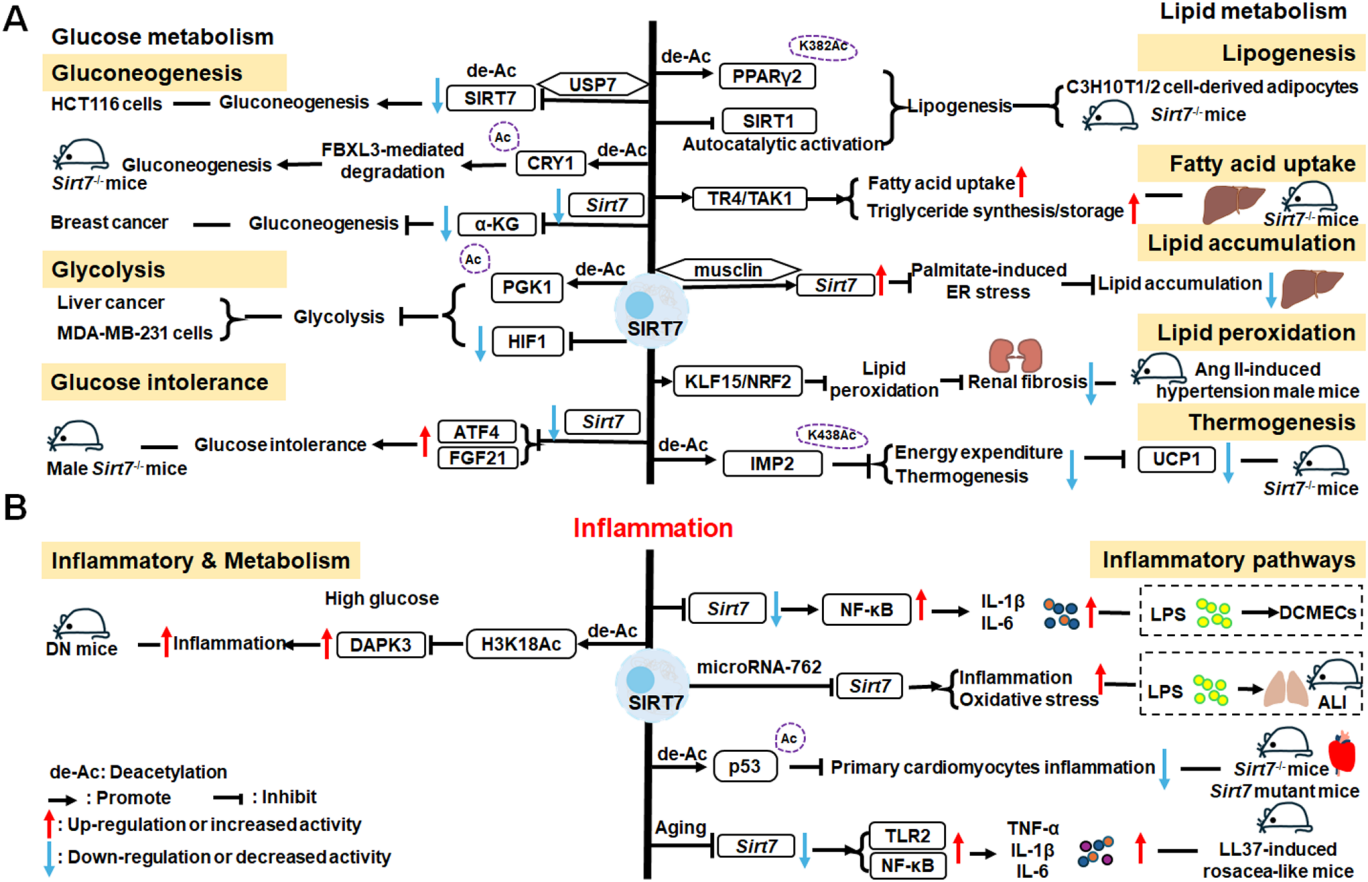
Overview of the roles of SIRT7 in metabolism and inflammation. **(A)** Effects of SIRT7 in glucose and lipid metabolism. SIRT7 controls gluconeogenesis, glycolysis, and glucose tolerance, and regulates lipid metabolism processes, including lipogenesis, fatty acid uptake, triglyceride synthesis/storage, lipid accumulation, and lipid peroxidation, as well as energy consumption and thermogenesis. **(B)** Effects of SIRT7 in regulating anti-inflammation processes in multiple contexts, mainly via the glucose metabolism, NF-κB and p53 pathways.

There is growing evidence that SIRT7 has significant roles in the regulation of metabolism and inflammation (**Figure 3**). SIRT7 has physiological functions in metabolic regulation that occur through a chromatin-dependent signaling pathway to maintain metabolic homeostasis (135). SIRT7 contributes to regulation of glucose and lipid metabolism by modulating various proteins in adipose tissue, liver, and kidney (54). For example, SIRT7 promotes gluconeogenesis, inhibits glycolysis, and maintains the balance between gluconeogenesis and glucose tolerance through its deacetylation activity. Moreover, SIRT7 exerts regulatory control over lipid metabolic processes through the testicular nuclear receptor 4 (TR4)/TAK1 pathway, the autophagy pathway, and the NRF2 pathway. This include promoting lipogenesis and fatty acid absorption while inhibiting lipid accumulation and peroxidation. In addition, it affects energy expenditure and thermogenesis. Additionally, SIRT7 modulates anti-inflammatory processes in multiple contexts, mainly through glucose metabolism, NF-κB and p53 pathways.

#### 2.3.1 Regulation of glucose metabolism

SIRT7 is associated with control of gluconeogenesis, glycolysis, and glucose tolerance, suggesting that it has roles in glucose metabolism (54). For example, the deacetylase activity of SIRT7 is negatively regulated by USP7 by removing the K63-linked polyubiquitin, thus decreasing the expression of *G6pc*, a gluconeogenic gene, and modulating gluconeogenesis in HCT116 cells (136). Hepatic SIRT7 deacetylates cryptochrome 1 (CRY1) at K565/579 to promote its F-box and leucine rich repeat protein 3 (FBXL3)-mediated degradation to regulate gluconeogenesis (137). Su et al. found that SIRT7 insufficiency reduces cellular α-ketoglutarate(α-KG), a metabolite product of isocitrate dehydrogenase 1 (IDH1), and suppresses gluconeogenesis (138). SIRT7 suppresses glycolysis by decreasing HIF-1 protein levels in MDA-MB-231 cells and deacetylating phosphoglycerate kinase 1 (PGK1) at the K323 site in liver cancer (139, 140). Besides, SIRT7 deficiency increases the levels of activating transcription factor 4 (*ATF4*) mRNA and enhanced fibroblast growth factor 21 (FGF21) expression, thus protecting against aging-associated glucose intolerance and extending lifespan in male mice, indicating that SIRT7 may play opposite roles in aging to those directed by SIRT1 and SIRT6 (141).

#### 2.3.2 Regulation of lipid metabolism

The roles of SIRT7 in the following aspects of lipid metabolism are discussed: lipogenesis, fatty acid uptake and triglyceride synthesis/storage, lipid accumulation, lipid peroxidation, energy consumption, and thermogenesis. SIRT7 regulates lipogenesis in adipocytes through deacetylation of PPARγ2 K382 in C3H10T1/2 cell-derived adipocytes (142). Additionally, SIRT7 inhibits autocatalytic activation of SIRT1 to promote lipogenesis in mice (142). In the liver, hepatic SIRT7 controls lipid metabolism by positively regulating TR4/TAK1, and increasing TR4 target gene levels, to enhance fatty acid uptake and triglyceride synthesis/storage (143). Musclin, a cytokine secreted by bone and skeletal muscle, can suppress palmitate-induced endoplasmic reticulum (ER) stress by upregulating SIRT7 and autophagy signaling, thereby alleviating lipid accumulation in primary hepatocytes (144). In renal tissue, SIRT7 alleviates renal cell ferroptosis, lipid peroxidation, and partial epithelial-mesenchymal transition under hypertensive conditions by facilitating the Kruppel-like factor 15 (KLF15)/NRF2 signaling, thereby mitigating renal fibrosis, injury, and dysfunction in hypertensive mice (145). In adipose tissue, SIRT7 suppresses energy expenditure and thermogenesis by deacetylating insulin-like growth factor 2 mRNA-binding protein 2 (IMP2) K438, and attenuating the levels of uncoupling protein 1 (UCP1) in adipose tissue-specific *Sirt7* KO (*Sirt7* AdKO) mice and brown adipose tissue specific *Sirt7* KO (*Sirt7* BAdKO) mice (146).

In summary, SIRT7 regulates various metabolic pathways through deacetylation of a number of target substrates. The development of inflammatory responses requires the participation of intracellular metabolism. In the subsequent section, we explore SIRT7-mediated inflammatory processes, while considering the potential involvement of metabolic pathways.

#### 2.3.3 SIRT7 mediated inflammation regulation

To date, few studies have focused on the details of SIRT7 mediated inflammatory responses through glucose or lipid metabolism. Li et al. discovered that SIRT7 regulates anti-inflammation via glucose metabolism in the occurrence of hyperglycemic memory in diabetic nephropathy (DN). High glucose-mediated mutual inhibition between SIRT7 and ETS domain-containing protein (ELK1) induced death-associated protein kinase-3 (DAPK3) transcription by deacetylating H3K18Ac and inflammation by modulating NF-κB pathway. Therefore, SIRT7 overexpression suppress DAPK3 levels and inflammation in glomerular endothelial cells (147).

SIRT7 participates in the pathogenesis of various immune-mediated inflammatory disorders (148); however, the inflammatory response of SIRT7 varies greatly in different situations, and mainly proceeds via the NF-κB and p53 pathways. Like other SIRTs, SIRT7 has significant roles in anti-inflammation processes. Chen et al. found that SIRT7 can regulate LPS-induced inflammatory injury by suppressing the NF-κB signaling pathway in dairy cow mammary epithelial cells (DCMECs) (80). Additionally, after LPS-induced ALI in mice, microRNA-762 increases inflammation, and oxidative stress through directly inhibiting *Sirt7* expression (149). Aging-conferred *Sirt7* depletion induced a rapid increase in the production of TNF-α, IL-6, and IL-1β by TLR2-NF-κB pathway in LL37-induced rosacea-like mice model (150), and *Sirt7* knockdown promotes nuclear translocation of NF-κB p-p65 (phosphorylated p65) and subsequently increases downstream inflammatory cytokine secretion, while SIRT7 overexpression has the opposite effect (80, 151). Additionally, SIRT7 deacetylates p53, increasing resistance to cytotoxic and oxidative stress in neonatal primary cardiomyocytes (152).

SIRT7 is among the least studied mammalian SIRTs to date (153), but is established to have an anti-inflammatory role through its deacetylation activity and the NF-κB and p53 pathways. Given reports of contradictory results, further studies are needed to clarify the roles of SIRT7 in inflammation-related cells.

## 3 SIRT2 is located in the cytoplasm and a key regulator of biological processes, such as metabolism and inflammation

### 3.1 SIRT2

**Figure 4:**
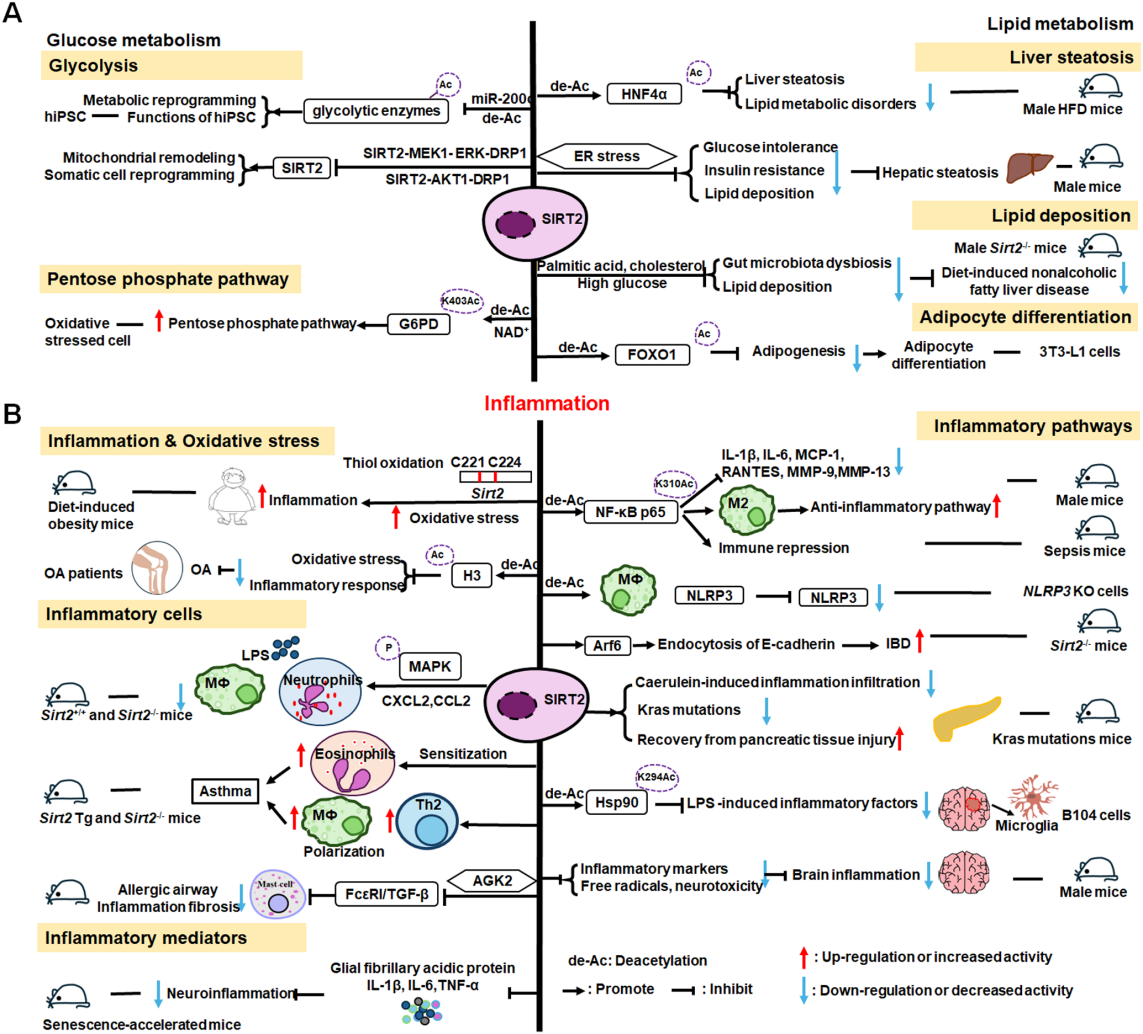
Overview of the roles of SIRT2 in metabolism and inflammation. **(A)** Effects of SIRT2 in glucose and lipid metabolism. SIRT2 mediates glucose metabolism through glycolysis and the pentose phosphate pathway, and engages in lipid metabolism processes, including steatosis, lipid deposition, and adipocyte differentiation. **(B)** Effects of SIRT2 in regulating inflammation, mainly focusing on oxidant stress, inflammatory cells, inflammatory factors, NF-κB, and NLRP3 pathways. SIRT2 modulates inflammation by influencing neutrophils, eosinophils, macrophages, and mast cells, and mainly exerts its anti-inflammatory effects by suppressing the expression of inflammatory factors, such as TNF-α, IL-6, and IL-1β. Additionally, SIRT2 exerts anti-inflammatory effects by inhibiting the NF-κB, NLRP3, and Arf6 pathways, among others.

The roles of SIRT2 in metabolism and inflammation are summarized in **Figure 4**. SIRT2 mediates glucose metabolism by inhibiting glycolysis during the reprogramming of induced pluripotent stem cells (iPSCs) and boosting the pentose phosphate pathway (PPP) via deacetylation activity. SIRT2 primarily inhibits lipid metabolism such as steatosis, lipid accumulation, and adipocyte differentiation. SIRT2 regulates inflammation mainly by focusing on oxidant stress, inflammatory cells, inflammatory factors and signaling pathways. SIRT2 modulates inflammation by influencing neutrophils, eosinophils, macrophages, and mast cells, and exerts its anti-inflammatory effects mainly by suppressing the expression of inflammatory factors. Additionally, SIRT2 exerts anti-inflammatory effects by inhibiting the NF-κB, NLRP3, and Arf6 pathways, among others.

#### 3.1.1 Regulation of glucose metabolism

SIRT2 mediates glucose metabolism through two pathways: glycolysis and PPP. Metabolic transition from mitochondrial oxidative phosphorylation to glycolysis is critical for somatic reprogramming of iPSCs (154), and SIRT2 regulates this transition by controlling the acetylation status of glycolytic enzymes (154–156). Numerous glycolytic enzymes, including aldolase, glyceraldehyde phosphate dehydrogenase (GAPDH), PGK1, enolase, and pyruvate kinases, are highly acetylated in human embryonic stem cells and pluripotent stem cells (154, 156). The deacetylase, SIRT2, is robustly downregulated in this process by miR-200c, impacting metabolic reprogramming and human iPSC function (156). Conversely, SIRT2 overexpression suppresses iPSC reprogramming (154). Cha et al. reported that SIRT2- mitogen-activated protein kinase kinase-1 (MEK1) - ERK- dynamin-related protein 1 (DRP1) and SIRT2-AKT1-DRP1 axes link SIRT2 downregulation to mitochondrial remodeling and somatic cell reprogramming (155); however, immune cell metabolic reprogramming through SIRT2 requires further study. In addition to regulating glycolysis, SIRT2 also controls the PPP. Glucose-6-phosphate dehydrogenase (G6PD) catalysis is the first step of the PPP, which regenerates the GSH antioxidant, NADPH, and generates ribose-5-phosphate, a substrate for NAD^+^ synthesis (157). SIRT2 mediates G6PD K403Ac deacetylation and activates G6PD via NAD^+^ to regulate PPP (157).

#### 3.1.2 Regulation of lipid metabolism

SIRT2 engages in liver disease progression by regulating lipid metabolism processes, such as steatosis, lipid deposition, and adipocyte differentiation. Ren et al. discovered that SIRT2 deacetylates HNF4α to prevent liver steatosis and lipid metabolic disorders (158); however, SIRT2 deficiency aggravates diet-induced nonalcoholic fatty liver disease by promoting gut microbiota dysbiosis and lipid deposition and changing the normal metabolites in the presence of palmitic acid, cholesterol, and high glucose (159). In mice, *Sirt2* deletion aggravates HFD-induced obesity, promotes glucose intolerance and insulin resistance, exacerbates hepatic steatosis, and increases lipid deposition via the ER stress pathway (160). SIRT2 is also involved in adipocyte differentiation; it is the most abundant SIRTs in adipocytes and reducing its expression promotes lipogenesis in 3T3-L1 cells to regulate adipocyte differentiation by increasing FOXO1 acetylation (161).

Hence, SIRT2 can regulate glycolysis, the PPP, and lipid metabolism; however, relatively few studies have investigated the role of SIRT2 in impacting inflammation via its influence on metabolic pathways.

#### 3.1.3 SIRT2 mediated inflammation regulation

Inflammation is an essential immune response, and SIRT2 is known to have anti-inflammatory properties (162). SIRT2 modulates sepsis inflammation and OA development in the context of obesity by inhibiting oxidative stress, while increased oxidative stress and cysteine (C221 and C224) thiol oxidation exacerbate hyper-inflammation (163). Further, SIRT2 upregulation can alleviate diabetic OA development by suppressing oxidative stress and inflammatory responses, likely related to H3K9,H3K14, and H3K56 deacetylation (164).

SIRT2 also has effects on inflammation processes in immune cells, such as neutrophils, eosinophils, macrophages, and mast cells. Jung et al. discovered that *SIRT2* deficiency ameliorates LPS-induced infiltration of neutrophils and macrophages, while regulating mitogen-activated protein kinase (MAPK) phosphatase-1 (MKP-1) acetylation-related p38/c-Jun N-terminal kinase (JNK) MAPK phosphorylation and expression of chemokine (C-X-C motif) ligand (CXCL) 2 and C-C motif chemokine ligand 2 (CCL2), thereby treating acute tubular injury (165). In allergic asthmatic inflammation, SIRT2 expression regulates eosinophil recruitment after sensitization (166). SIRT2 also aggravates asthmatic inflammation by up-regulating T-helper type 2 responses and macrophage polarization; however, an inhibitor of SIRT2, AGK2, ameliorates mast cell-mediated allergic airway inflammation and fibrosis by inhibiting the FcϵRI (a high-affinity IgE receptor)/TGF-β signaling pathway (167).

SIRT2 mainly exerts anti-inflammatory effects by suppressing inflammatory factors, such as TNF-α, IL-6, and IL-1β, in response to disease development. For example, early SIRT2 inhibition can prevent neuroinflammation evidenced by reduced levels of glial fibrillary acidic protein, TNF-α, IL-6, and IL-1β (168). Further, SIRT2 inhibits LPS-stimulated TNF-α production to suppress neuroinflammation (169, 170).

SIRT2 primarily exerts its anti-inflammatory effects by inhibiting the NF-κB, NLRP3, and Arf6 pathways, among others. For example, SIRT2 suppresses inflammatory responses in collagen-induced arthritis by deacetylating K310 of NF-κB p65, leading to reduced expression of NF-κB-dependent genes, including those encoding IL-1β, IL-6, monocyte chemoattractant protein 1 (MCP-1), matrix metalloproteinase 9 (MMP-9), and MMP-13 (171). Also, Sirt2 deletion promotes inflammatory responses by increasing NF-κB acetylation and by reducing the M2-associated anti-inflammatory pathway (172). Similarly, in obesity with sepsis, Sirt2 expression and activity decrease during hyper-inflammation, while during hypo-inflammation, total *Sirt2* expression increases, and SIRT2 deactivates NF-κB to mediate immune repression in mice (162). In addition to the NF-κB pathway, SIRT2 exerts anti-inflammatory effects in the NLRP3 pathway. For example, SIRT2 deacetylates NLRP3 in macrophages and inactivates the NLRP3 inflammasome, which functions to reverse aging-associated inflammation and insulin resistance (173). SIRT2 inhibits inflammatory bowel disease by regulating the gut epithelium barrier via inhibition of Arf6-mediated endocytosis of E-cadherin, a protein important for intestinal epithelial integrity (174). Sirt2 deficiency increases inflammatory infiltration during caerulein-induced acute pancreatitis and strongly impairs recovery from pancreatic tissue injury, as well as leading to accumulation of oncogenic Kras mutations in the pancreas (175). Heat-shock protein 90 (Hsp90) K294 is deacetylated by SIRT2, and SIRT2 suppresses LPS-induced inflammatory factor expression via Hsp90-glucocorticoid receptor signaling in B104 cells (176). SIRT2 is a suppressor of brain inflammation, and reduction of SIRT2 levels increases the expression of inflammatory markers, the production of free radicals, and neurotoxicity (177).

Research on SIRT2 and its role in inflammation is abundant; however, the specific inflammatory effects of SIRT2 warrant further exploration. Existing compounds targeting SIRT2 may impact inflammatory cell metabolism and reprogramming by achieving substrate-selective activation or inhibition, with important implications for the direction and development of new disease therapies.

## 4 Besides glucose and lipid metabolism, the mitochondria-located SIRTs, SIRT3, SIRT4, and SIRT5, contribute to regulation of amino acid, particularly glutamine metabolism

### 4.1 SIRT3

**Figure 5:**
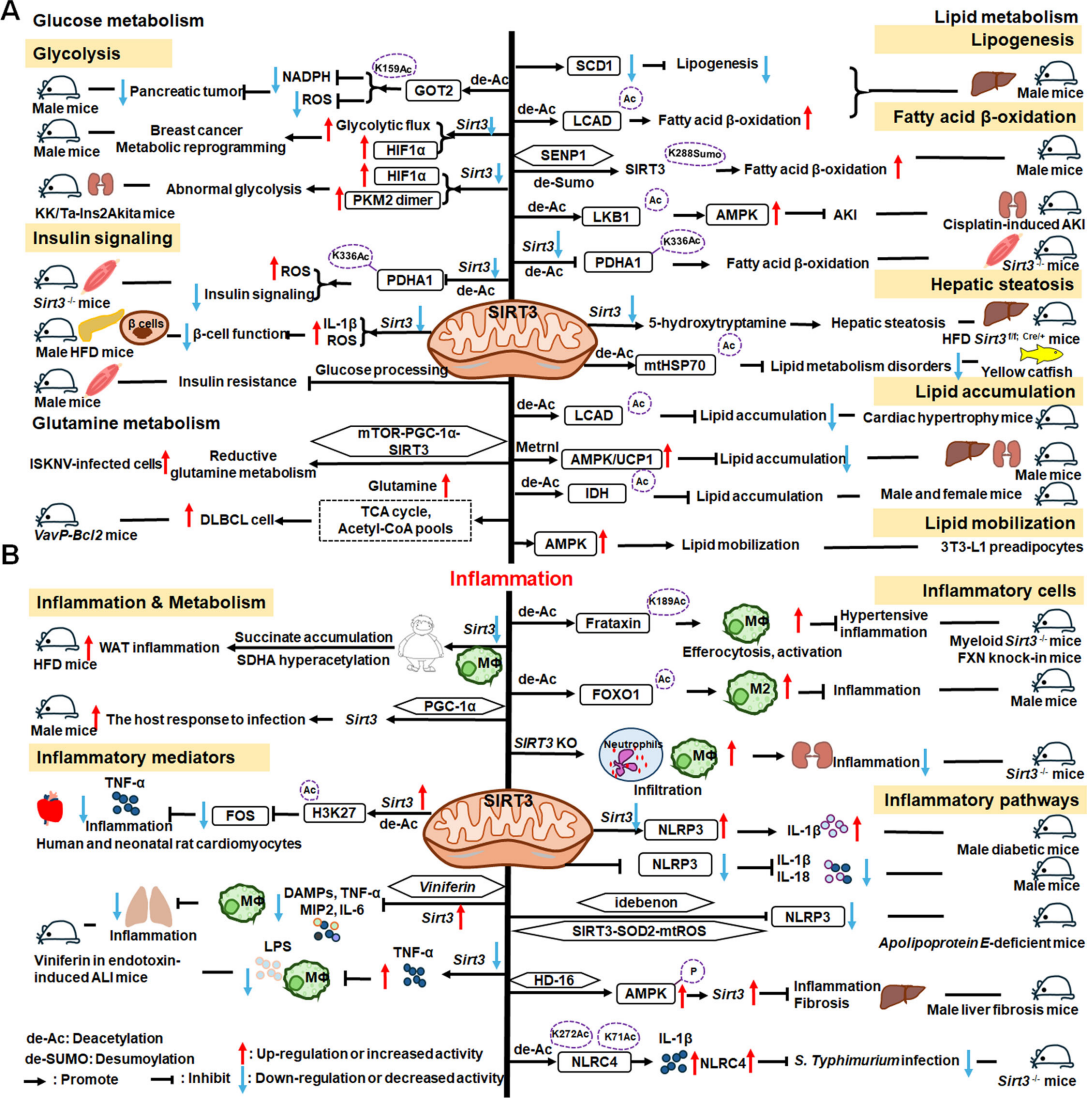
Overview of the roles of SIRT3 in metabolism and inflammation. **(A)** Effects of SIRT3 in glucose, lipid, and glutamine metabolism. The roles of SIRT3 roles in glucose metabolism focuses on glycolysis and insulin signaling. SIRT3 also controls lipogenesis, fatty acid β-oxidation, hepatic steatosis, lipid accumulation, and lipid mobilization. In addition to glucose and lipid metabolism, SIRT3 plays a key role in glutamine metabolism. **(B)** Effects of SIRT3 in regulating inflammation. The PGC-1α pathway is important in SIRT3 regulation of infection. SIRT3 also impacts inflammation in macrophages and neutrophils and modulates the expression of inflammatory factors, including TNF-α, DAMPs, MIP2, and IL-6. Moreover, NLRP3 and NLRC4 are the main pathways regulating inflammation via SIRT3.

As illustrated in **Figure 5**, SIRT3 has key roles in metabolism and inflammation. SIRT3 is central to the control of metabolic processes, including glucose, lipid, and glutamine metabolism (178). Through its deacetylation and regulation of mitochondrial functions, SIRT3 plays a key role in glucose metabolism, with a focus on inhibiting glycolysis and maintaining the balance of insulin signaling. SIRT3 also controls lipid metabolism, including suppressing lipogenesis and lipid accumulation, as well as promoting fatty acid β-oxidation, hepatic steatosis, and lipid mobilization. In addition to glucose and lipid metabolism, SIRT3 participates in glutamine metabolism via the PGC-1α pathway and TCA cycle. Furthermore, SIRT3 regulates inflammation mainly by some metabolic pathways. SIRT3 also influences inflammation in macrophages and neutrophils and modulates the expression of inflammatory factors by deacetylating the associated proteins. Additionally, NLRP3, AMPK and NLRC4 are the main pathways that regulate inflammation via SIRT3.

#### 4.1.1 Regulation of glucose metabolism

Regarding the function of SIRT3 glucose metabolism, in this paper, we focus on its roles in glycolysis and insulin signaling. The malate-aspartate shuttle is indispensable for the net transfer of cytosolic NADH into mitochondria, to maintain high glycolysis rates and support rapid tumor cell growth. SIRT3-dependent glutamic-oxaloacetic transaminase 2 (GOT2) K159Ac status affects malate-aspartate NADH shuttle activity and pancreatic tumor growth (179). Similarly, loss of Sirt3 also drives metabolic reprogramming and the development of breast cancer via stabilizing HIF-1α and increasing glycolytic flux (180). In a mouse model of progressive diabetic kidney disease (the KK/Ta-Ins2Akita mouse), *Sirt3* deficiency leads to abnormal glycolysis through regulating HIF-1α and PKM2 dimer accumulation, leading to diabetic renal fibrosis (181). Besides, oxygen consumption and oxidative stress in muscle cells of *Sirt3^-/-^* mice are decreased, impairing glucose oxidation, ROS level, and insulin signaling by deacetylating PDH E1α subunit (PDHA1) K336Ac and downregulating the activity of PDHA1 (182, 183). In islets isolated from patients with type II diabetes and mice islets or INS1 cells treated with IL-1β and TNF-α, SIRT3 expression is markedly downregulated, which impairs β-cell function via abnormal elevation of ROS levels and amplification IL-1β synthesis (184). In addition, SIRT3 prevents dietary insulin resistance by promoting muscle glucose processing and mitochondrial function in male mice (185).

#### 4.1.2 Regulation of lipid metabolism

SIRT3 is a mitochondrial NAD^+^-dependent deacetylase that controls the acetylation status of many enzymes and proteins involved in energy metabolism, impacting lipogenesis, fatty acid β-oxidation, hepatic steatosis, lipid accumulation, and lipid mobilization. For example, SIRT3 enhances mitochondrial energy production to suppress lipogenesis by decreasing stearoyl-CoA desaturase 1 (SCD1) production (186). Further, SIRT3 promotes fatty acid β-oxidation by reducing long-chain acyl CoA dehydrogenase (LCAD) acetylation levels in male mice (186, 187). SENP1-mediated desumoylation of SIRT3 in mitochondria regulates levels of protein deacetylation and mitochondrial metabolism, including fatty acid oxidation (188). SIRT3 also modulates fatty acid oxidation by deacetylating liver kinase B1 (LKB1) and activating AMPK to improve cisplatin-induced acute kidney injury (AKI) in mice (189). *Sirt3* deletion decreases the activity of the PDHA1 in muscle cells and reduces glucose oxidation, leading to fatty acid oxidation (183). Ming et al. discovered that *Sirt3* deficiency in the pancreas regulates insulin secretion and increases hepatic steatosis by improving 5-hydroxytryptamine synthesis in *Sirt3* ^f/f; Cre/+^ mice under the HFD condition (190). Besides, choline can alleviate HFD-induced hepatic lipid metabolism disorders through SIRT3-mediated mitochondrial HSP70 deacetylation (191). SIRT3 reduces lipid accumulation by deacetylating LCAD in a mouse model of cardiac hypertrophy (192). Metrnl, a cytokine in the kidney, regulates kidney metabolism to maintain cellular mitochondrial homeostasis and alleviate lipid accumulation by upregulating SIRT3-AMPK/UCP1 signaling in renal tubular epithelial cells in male mice (193); the regulation of lipid accumulation via SIRT3-AMPK/UCP1 signaling pathway is also found in human hepatic cells (194). Further, SIRT3 overexpression suppresses lipid accumulation in macrophages via decreasing mitochondrial IDH2 acetylation levels (195). In addition, SIRT3 promotes lipid mobilization in adipocytes by activating AMPK (196).

#### 4.1.3 Regulation of glutamine metabolism

SIRT3 also plays a key role in glutamine metabolism, and SIRT3-mediated reductive glutamine metabolism may function in antioxidant stress and contribute to survival of cells infected with spleen and kidney necrosis viruses (ISKNV) via the mTOR-PGC-1α-SIRT3 pathway (197). Diffuse large B cell lymphomas (DLBCLs) are dependent on SIRT3 for proliferation, survival, self-renewal, and tumor growth *in vivo*. Sirt3 depletion induces DLBCL cell death by reducing glutamine flux to the TCA cycle and acetyl-CoA pools in *VavP-Bcl2* mice (198).

Therefore, SIRT3 is an important drug target in metabolic diseases. To further explore the relationships between metabolism and inflammation, investigations of how SIRT3 affects metabolism in inflammatory cells are warranted.

#### 4.1.4 SIRT3 mediated inflammation regulation

SIRT3 regulation of lipid metabolism can occur in macrophages, indicating that metabolism and inflammatory cells are connected, which can inform deeper understanding of the relationship between metabolism and inflammation. Loss of Sirt3 exacerbates HFD-induced obesity and mitochondrial dysfunction, as well as promoting white adipose tissue (WAT) inflammation via succinate accumulation and SDHA hyperacetylation in mice (199). Further, the PGC-1α-SIRT3 mitochondrial pathway can attenuate potentially damaging inflammasome activation and augment the host response to infection in male mice (200).

SIRT3 has important roles in inflammatory processes involving inflammatory cells, mediators, and pathways. For example, SIRT3 enhances efferocytosis and pro-inflammatory macrophage activation by deacetylating Frataxin (FXN) K189 in myeloid *Sirt3^-/-^* mice and FXN knock-in mice (201). Moreover, SIRT3 overexpression in macrophages induces M2 macrophage polarization and alleviates inflammation by deacetylating FOXO1 (202). *Sirt3^-/-^* mice exhibit significantly increased infiltration of inflammatory cells, including neutrophils and macrophages, as well as monocyte chemoattractant protein-1 (MCP-1) expression in the context of cisplatin−induced renal inflammation (203).

Overexpressed or activated *Sirt3* can reduce inflammatory responses through anti-inflammatory mediators, such as TNF-α. In human and neonatal rat cardiomyocytes, *Sirt3* overexpression partially prevents TNF-α-induced inflammatory and profibrotic responses by inhibiting *FOS* transcription through deacetylation of the specific histone, H3K27, in its promoter region (204). Moreover, Kurundkar et al. found that *Sirt3* deficiency altered macrophage proinflammatory responses to LPS, by increasing TNF-α production (205). *Viniferin*, a specific SIRT3 activator, modulates the production of DAMPs and proinflammatory cytokines, including TNF-α, IL-6, and macrophage inflammatory protein (MIP)-2, as well as inflammasome activation in macrophages (205).

SIRT3 also regulates inflammation via the NLRP3, AMPK, and nucleotide-binding oligomerization domain, leucine-rich repeat and caspase recruitment domain-containing 4 (NLRC4) pathways. *Sirt3* deficiency can promote NLRP3 activation in myocardial cells stimulated by high glucose in diabetic mice, thus aggravating diabetic heart disease (206). Zhao et al. suggested that SIRT3 plays a protective role against mitochondrial damage in the kidney by attenuating ROS production, inhibiting the NRLP3 inflammasome, and downregulating IL-1β and IL-18 (207). Small molecules are reported to regulate inflammatory processes involving SIRT3. Idebenone, a short-chain benzoquinone, similar in structure to coenzyme Q10, protects against atherosclerosis in apolipoprotein E-deficient mice by clearing oxygen free radicals and inhibiting NLRP3 activation via the SIRT3/superoxide dismutase 2 (SOD2)/mitochondrial ROS pathway (208). Additionally, Li et al. found that hesperetin derivative-16, a monomer compound with anti-inflammatory and hepatoprotective effects, can attenuate CCl_4_-induced liver inflammation and fibrosis by activating the AMPK/SIRT3 pathway (209). Further, SIRT3-mediated deacetylation of NLRC4 at K71 or K272 promotes NLRC4 inflammasome assembly, thereby increasing inflammasome activation and IL-1β production (210).

In conclusion, SIRT3 can mediate inflammatory responses by regulating the PGC-1α pathway and is widely involved in inflammatory response via inflammatory cells (macrophages and neutrophils), mediators (TNF-α, DAMPs, MIP2 and IL-6), and pathways (NLRP3 and NLRC4 pathways). Further in-depth studies are needed to identify and elucidate the exact roles of SIRT3 in inflammation via metabolic pathways.

### 4.2 SIRT4

**Figure 6:**
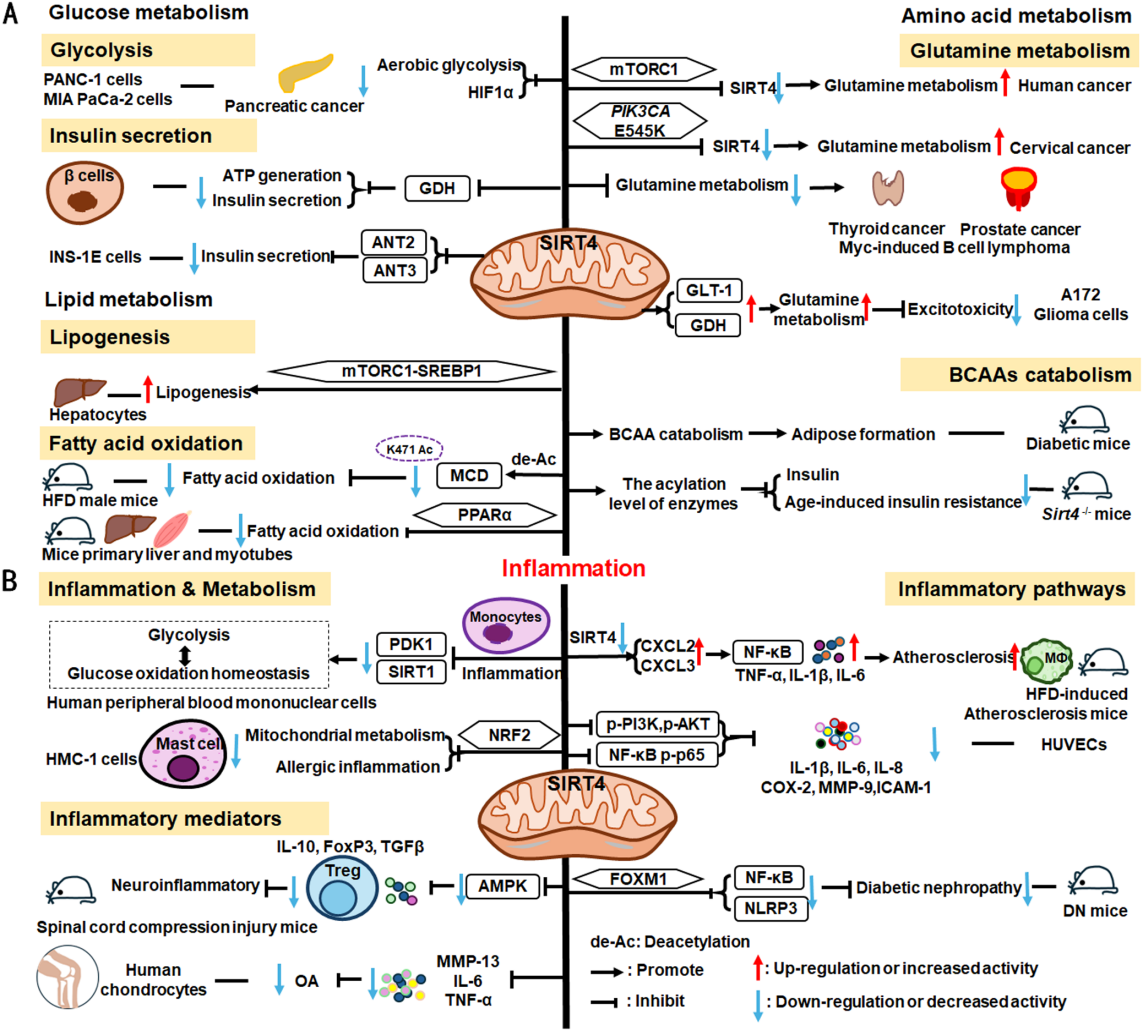
Overview of the roles of SIRT4 in metabolism and inflammation. **(A)** Effects of SIRT4 in glucose, lipid, and amino acid metabolism. SIRT4 regulates glucose metabolism mainly through its roles in glycolysis and insulin secretion. Moreover, SIRT4 is an important regulator of lipid, glutamine, BCAA metabolism. **(B)** Effects of SIRT4 in regulating inflammation through its effects on glycolysis, the NRF2 pathway, inflammatory mediators, and inflammatory pathways, via NF-κB and NLRP3 signaling.

SIRT4 is another member of the SIRTs that localizes to the mitochondrial matrix (211). The key roles of SIRT4 in metabolism and inflammation are summarized in **Figure 6**. SIRT4 regulates glucose metabolism mainly through inhibiting glycolysis and insulin secretion via the HIF-1α pathway, as well as regulating the activity of several enzymes. Moreover, SIRT4 is an important negative regulator of lipid metabolism, promoting the lipogenesis and inhibiting fatty acid oxidation. Furthermore, SIRT4 regulates the expression and acylation of enzymes, which is important for glutamine and leucine metabolism. Additionally, SIRT4 mainly regulates anti-inflammation through its effects on glycolysis, the NRF2 pathway, inflammatory mediators, and inflammatory pathways (e.g., NF-κB and NLRP3 signaling pathways).

#### 4.2.1 Regulation of glucose metabolism

SIRT4 regulates glucose metabolism mainly through its roles in glycolysis and insulin secretion. SIRT4 negatively regulates Warburg effects, such as inhibiting aerobic glycolysis, tumor growth and suppressing HIF-1α level in PANC-1 and MIA PaCa-2(pancreatic cancer cell lines) (212). Studies have shown that SIRT4 regulates the secretion of insulin. Haigis et al. reported that SIRT4 is highly abundant in the mitochondrial matrix of pancreatic β-cells, which can downregulate glutamate dehydrogenase (GDH) activity. Mechanically, SIRT4 decreases mitochondrial fuel oxidation into the TCA cycle, leading to a decreased ATP/ADP ratio, which influences ATP generation and impairs insulin secretion (213). In addition to GDH, Nidhi and colleagues identified that SIRT4 targets insulin-degrading enzymes and adenine nucleotide translocator 2 (ANT2) and ANT3 to regulate insulin secretion in INS-1E cells (214). And loss of SIRT4 expression in specific organs and cells, such as pancreatic β cells, leads improved insulin secretion in response to glucose (214, 215).

#### 4.2.2 Regulation of lipid metabolism

SIRT4 is an important regulator of lipid metabolism, primarily through its functions in lipogenesis and fatty acid oxidation. SIRT4 controls lipogenesis via the mTOR complex 1 (mTORC1)-SREBP1 pathway and activates the lipogenesis response in hepatocytes (216). In nutrient-rich conditions, SIRT4 represses fatty acid oxidation through deacetylating malonyl CoA decarboxylase (MCD) K471 and inhibiting the activity of MCD (216, 217). Moreover, SIRT4 represses PPARα activity to suppress hepatic fat oxidation (218). However, the expression of mitochondria and fatty acid metabolic enzymes was significantly increased in primary hepatocytes and myotubes of *Sirt4^-/-^* mice, improving fatty acid oxidation (219).

#### 4.2.3 Regulation of amino acid metabolism, particularly glutamine metabolism

The mTORC1 pathway represses SIRT4 by promoting proteasome-mediated destabilization of CREB2, which stimulates glutamine metabolism and cell proliferation (220). SIRT4 is thought to act as a tumor suppressor by repressing glutamine metabolism in Myc-induced B cell lymphoma (221). Further, in cervical cancer, the *PIK3CA* mutation, E545K, negatively regulates SIRT4 expression via the epigenetic regulator, EP300, independently of the canonical mTORC1 pathway, thus reducing radiosensitivity by promoting glutamine metabolism (222). SIRT4 also inhibits glutamine metabolism to regulate thyroid cancer cell proliferation and migration (223), and acts as a tumor suppressor in prostate cancer by the same mechanism (224); however, in A172 glioma cells, SIRT4 up-regulates glutamate metabolism to prevent excitotoxicity by increasing glutamate transporter 1 (GLT-1) and GDH expression and decreasing glutamine synthetase levels (225).

In addition to glutamine metabolism, SIRT4 also regulates the catabolism of BCAAs, which are the most consumed metabolites in differentiating adipocytes (226). Mitochondrial SIRT4 promotes BCAAs catabolism and adipose formation; however, SIRT4-mediated BCAA catabolism is down-regulated in the adipose tissue of diabetic mice (226). Moreover, Kristin and colleagues found that SIRT4 could control leucine metabolism through regulating the acylation levels of enzymes in the pathway, and mice lacking *Sirt4* have dysregulated leucine metabolism that can lead to increased insulin and development of age-induced insulin resistance (227).

Overall, SIRT4 regulates glucose metabolism, including the inhibition of glycolysis and insulin secretion. SIRT4 is also an important regulator of lipid metabolism, including lipogenesis and fatty acid oxidation. Moreover, SIRT4 regulates glutamine and BCAA metabolisms; however, only a few reports have explored how SIRT4 can mediate inflammation through its regulation of metabolism. Thus, further in-depth studies are needed to identify and elucidate the exact role of SIRT4 in inflammation.

#### 4.2.4 SIRT4 mediated inflammation regulation

SIRT4 is reported to mediate inflammation through its functions in metabolic pathways. For example, SIRT4 can repress pyruvate dehydrogenase kinase 1 (PDK1) expression during the monocyte acute inflammatory response, by rebalancing glycolysis and glucose oxidation homeostasis in human peripheral blood mononuclear cells (228). Glutamine is a necessary metabolite for inflammatory factor synthesis and secretion (229). Hu et al. have confirmed that in mast cells (HMC-1 cells), SIRT4 overexpression significantly inhibits mitochondrial metabolism (mainly glutamine metabolism), mast cell degranulation, and allergic inflammation via the NRF2 pathway (229).

SIRT4 can regulate anti-inflammatory processes by affecting inflammatory cells, the expression of inflammatory factors, and pathways. In neuroprotective immune responses, Tregs increase secretion of the more anti-inflammatory factors, IL-10, FoxP3, and TGF-β, while SIRT4 suppresses the anti-neuroinflammatory activity of Tregs infiltrating the spinal cord following injury by decreasing the phosphorylation of AMPKα and blocking the AMPK pathway (230). Furthermore, SIRT4 mediates anti-inflammatory factor expression. For example, SIRT4 prevents OA development by suppressing inflammatory factors, including MMP-13, IL-6, and TNF-α in chondrocytes (231). SIRT4 also exerts anti-inflammatory effects by modulating inflammatory pathways, such as NF-κB, NLRP3, and NRF2 signaling. Loss of *Sirt4* enhances atherosclerosis development and inflammation through the NF-κB/IκB/CXCL2/3 pathway in mice (232). Also, overexpression of Sirt4 enhances HUVEC survival and inhibits inflammatory cytokine levels by suppressing PI3K phosphorylation (p-PI3K) and phosphorylated (p)−AKT, NF−κB p−p65 (233). Additionally, SIRT4 suppresses inflammatory responses by blocking the NF-κB pathway and decreasing the levels of pro-inflammatory cytokines (IL-1β, IL-6, and IL-8), COX-2, MMP-9, and ICAM-1 in HUVECs (234). Besides, Forkhead box M1 (FOXM1)-activated SIRT4 inhibits NF-κB signaling and the NLRP3 inflammasome to alleviate kidney injury and podocyte pyroptosis in DN mice (235).

To summarize, SIRT4 can mediate inflammation through rebalancing glycolysis and glucose oxidation homeostasis. Few studies have investigated the role of SIRT4 in inflammation, and these have mainly focused on its effects on the secretion of inflammatory factors and regulation of inflammatory pathways. For example, SIRT4 can suppress inflammatory factors, including MMP-13, IL-6, TNF-α, and TGF-β, among others. SIRT4 also participates in inflammatory responses by regulating the NF-κB, NLRP3, and NRF2 pathways. In subsequent studies, more attention should be paid to how SIRT4 participates in the metabolic responses of inflammatory cells, to control the occurrence of, and processes that contribute to, inflammatory responses.

### 4.3 SIRT5

**Figure 7:**
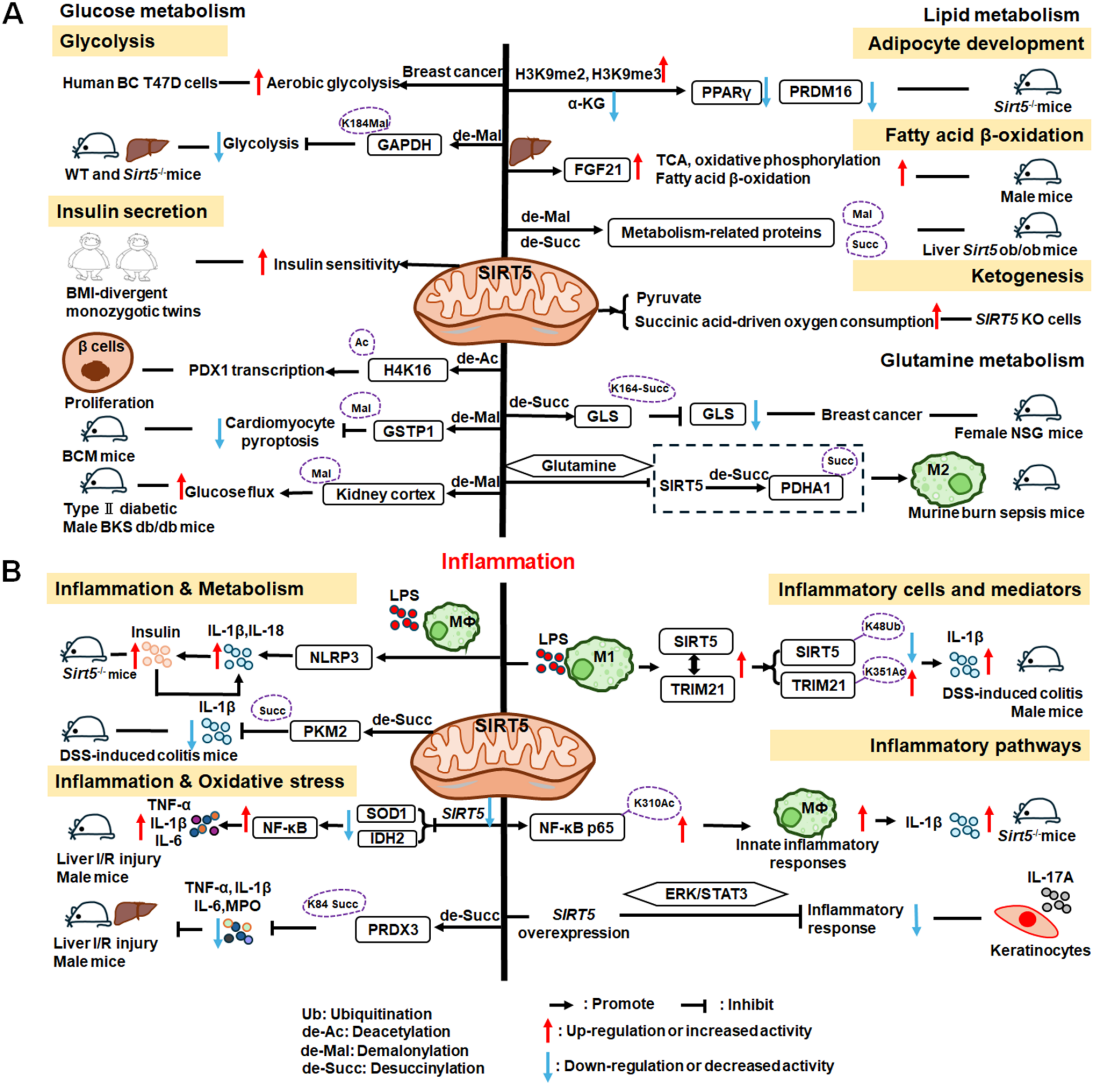
Overview of the roles of SIRT5 in metabolism and inflammation. **(A)** Effects of SIRT5 in metabolism. SIRT5 is mainly involved in glucose metabolism through its roles in glycolysis and insulin secretion. SIRT5 plays an important role in lipid metabolism, including adipocyte development, fatty acid β-oxidation, and ketogenesis. SIRT5 can also regulate glutamine metabolism through its effect of desuccinylation. **(B)** Effects of SIRT5 in regulating inflammation, including insulin secretion, PKM2 pathway, oxidative stress, inflammatory cells and mediators, as well as the NF-κB and STAT3 pathways.

SIRT5 plays significant roles in metabolism and inflammation (**Figure 7**). Specifically, in addition to acetylation modification, SIRT5 regulates a series of newly discovered acyl modifications, including succinylation, malonylation, and glutarylation. SIRT5 has major roles in regulating various metabolic pathways, including glucose, lipid, and glutamine metabolism. SIRT5 is mainly involved in glucose metabolism as it plays a role in deacetylation and demalonylation in glycolysis and insulin secretion. SIRT5 is an important positive regulator in lipid metabolism, including adipocyte development, fatty acid β-oxidation, and ketogenesis. SIRT5 can also regulate glutamine metabolism via its desuccinylation. Furthermore, SIRT5 regulates inflammation through glucose metabolism, PKM2 pathway, inflammatory cells, and mediators, as well as the NF-κB and STAT3 pathways.

#### 4.3.1 Regulation of glucose metabolism

The functions of SIRT5 in glucose metabolism primarily involve two pathways: glycolysis and insulin secretion. He et al. discovered that SIRT5 promotes aerobic glycolysis during BC development and metastasis in human BC T47D cells (236), while Nishida et al. found that SIRT5 regulates the glycolysis pathway via GAPDH K184 demalonylation and modulates its enzymatic activity in mice liver (237). Besides, in adipose tissue, SIRT5 levels are positively correlated with insulin sensitivity and negatively associated with inflammation in body mass index (BMI)-divergent monozygotic twins (238). Therefore, SIRT5 also plays a role in diabetes and related diseases. Liu et al. discovered that SIRT5 deglycosylated PDHA1 at K351 and increased the activity of pyruvate dehydrogenase complex, thereby altering the metabolic crosstalk with the TCA cycle and inhibiting the Warburg effect (239). Moreover, SIRT5 may regulate the transcription of pancreatic and duodenal homeobox 1 (*PDX1*) via H4K16 deacetylation, to participate in pancreatic β−cell proliferation and insulin secretion in type II diabetes (240). In diabetic cardiomyopathy(DCM), SIRT5 suppresses cardiomyocyte pyroptosis via lysine demalonylation of glutathione S-transferase P (GSTP1) in DCM mice (241), while in diabetic kidney disease, SIRT5 may regulate non-mitochondrial ATP production by decreasing malonylation in the kidney cortex of type II diabetic male BKS *db/db* mice (242).

#### 4.3.2 Regulation of lipid metabolism

SIRT5 plays important roles in adipocyte development, fatty acid β-oxidation, and ketogenesis. First, SIRT5, which links cellular metabolism to adipocyte development, promotes the levels of brown adipogenic regulators, such as PPARγ and PR domain-containing 16 (PRDM16), by increasing intracellular α-KG concentrations (243). Moreover, Zhou et al. found that hepatic overexpression of Sirt5 reduced myocardial infarction and cardiac fibrosis, promoted endocrine FGF21 release from the liver, and improved cell metabolism, by increasing levels of proteins regulating the TCA cycle, oxidative phosphorylation, and fatty acid β-oxidation pathways (244). Further, elevated Sirt5 expression improves the metabolic abnormalities in the liver of ob/ob mice by demalonylating and desuccinylating metabolism-related proteins (245). In addition to fatty acid β-oxidation, ketogenesis is an enriched lysine succinylation pathway targeted by SIRT5 (211). Specifically, Park et al. discovered that *SIRT5* KO cells exhibit elevated pyruvate and succinic acid-driven oxygen consumption (246).

#### 4.3.3 Regulation of glutamine metabolism

Similar to the roles of SIRT3 and SIRT4 in mitochondria, SIRT5 can regulate glutamine metabolism, in addition to participating in glucose and lipid metabolism. SIRT5 regulates glutamine metabolism in transformed cells by mediating desuccinylation of glutaminase (GLS) K164, thereby protecting GLS from ubiquitination at K158 and from subsequent degradation in female NOD scid gamma (NSG) mice (247). Additionally, Zhu et al. discovered that glutamine alleviates inflammatory injury in murine burn sepsis by inhibiting SIRT5-dependent desuccinylation of PDHA1, to maintain PDH activity and promote M2 macrophage polarization (248); hence, glutamine metabolism appears be associated with SIRT5 and macrophage polarization. These findings indicate that SIRT5 may have additional intersections with inflammatory cells through metabolic pathways; for example, by affecting the secretion of inflammatory factors.

#### 4.3.4 SIRT5 mediated inflammation regulation

SIRT5 can modulate inflammation by regulating metabolic processes, such as insulin secretion, PKM2, and mitochondrial oxidative stress. SIRT5 has a potential role in regulating insulin secretion and glucose homeostasis. *Sirt5* deficiency boosts IL-1β and IL-18 production by modulating the NLRP3 inflammasome in LPS treated macrophages in *Sirt5*-deficient (*Sirt5* ^−/−^) mice (249). Furthermore, increased IL-1β enhanced the secretion of insulin, which binds to insulin receptor (InsR) on macrophages and leads to increased AKT and JNK phosphorylation as well as IL-1β (249). Hence, IL-1β and insulin synergistically regulate glucose homeostasis for bacterial infections (249). The interplay between metabolic reprograming and inflammation also was highlighted in PKM2 pathway. For instance, SIRT5 participates in the inflammatory response in macrophages by desuccinylating and activating PKM2 to block macrophage IL-1β production and prevent DSS-induced colitis in mice (250). During hepatic I/R injury, SIRT5 was downregulated and decreased superoxide dismutase (SOD1) and IDH2 expression to inhibit its inhibitory effect on oxidative stress and inflammation by NF-κB pathway (251). Moreover, SIRT5 can ameliorate apoptosis and promote peroxiredoxin 3 (PRDX3) desuccinylation at K84 to activate PRDX3, which attenuates the inflammation response and downregulates TNF-α, IL-1β, IL-6 and myeloperoxidase (MPO) levels, to inhibit hepatic I/R injury in male mice (252).

Furthermore, SIRT5 regulates anti-inflammation by inflammatory cells. After pro-inflammatory macrophage polarization induced by LPS, the interaction between tripartite motif-containing 21 (TRIM21) and SIRT5 is enhanced, promoting SIRT5 K48-ubiquitination and degradation and sustaining TRIM21 K351Ac levels, thereby enhancing IL-1β expression (253). Additionally, NF-κB and STAT3 signaling are two major inflammatory response pathways in which SIRT5 participates. SIRT5 and SIRT1/2 have opposite expression patterns and functions in macrophages during sepsis. Competing with SIRT2, SIRT5 enhances the acetylation (K310) of p65 and activates the NF-κB pathway and its downstream cytokines, such as IL-1β,TNF-α and MCP-1, as well as enhances innate immune responses in endotoxin-tolerant macrophages (254). In IL-17A-stimulated psoriatic epidermal keratinocytes, SIRT5 reduces the inflammatory response via the ERK/STAT3 pathway, such as decreasing IL-8, IL-1β, IL-6 levels and ameliorating barrier dysfunction (255).

In summary, there is little research on the role of SIRT5 in inflammation. First, SIRT5 can modulate inflammation by regulating metabolism. SIRT5 participates in the inflammatory response in macrophages, where the main inflammatory factor responsible is IL-1β. Additionally, SIRT5 often modulates inflammatory responses via the NF-κB and STAT3 pathways. Further in-depth studies are needed to identify and elucidate the exact role of SIRT5 in inflammation via metabolic pathways, which will provide potential targets in metabolic and inflammatory diseases.

## 5 Inflammation regulation orchestrated by SIRTs

All SIRTs are involved in glucose and lipid metabolism; however, different SIRTs exhibit distinct subcellular localizations and varying preferences for metabolic processes (**Table 1**). This observation also suggests that different SIRTs may impact inflammation or cytokine secretion. For example, SIRT1, 2, 6, and 7 participate in glucose and lipid metabolism, while the mitochondrial SIRTs not only modulate glucose and lipid metabolism, but also regulate amino acid metabolism. Additionally, SIRT4 is involved in regulating BCAA metabolism. The expression/activity of SIRTs generally maintains a balance between glucose and lipid metabolism, thereby supporting cellular immunometabolism homeostasis (256).

**Table 1:** The roles of different SIRTs in various metabolic pathways.

SIRTs	Metabolic pathway		Mechanism	Function
SIRT1	Glucose metabolism	Gluconeogenesis	CREB deacetylation (55)	Inhibit
			PGC-1α and FOXO1deacetylation (56, 57)	Enhance
		Glycolysis	PGC-1α deacetylation (57)	Inhibit
			HIF-1α K674 deacetylation (59)	
		Insulin resistance	Upregulate AMPK (30)	Maintain homeostasis
			SIRT1-L107P enhances hyperinflammation and metabolic dysfunction (60)	
			Anti-inflammatory effects of 3T3-L1 adipocytes (62)	
	Lipid metabolism	Lipid homeostasis	Regulate PPAR-α and fatty acid metabolism (65)	Maintain homeostasis
		Lipogenesis	Inhibit PPAR-γ by binding to NCoR and SMRT (67)	Inhibit
		Lipolysis		Enhance
		Lipid accumulation	Block PPAR-γ and adipocyte differentiation (68)	Inhibit
		Liver metabolic damage	Decreased SREBP-1 expression and improved antioxidant and anti-inflammatory status (66)	Maintain homeostasis
		Alcoholic fatty liver	Lipin-1β/α signaling (64)	Enhance
		Nonalcoholic fatty liver disease	SIRT1/AMPK and NF-κB signaling pathways (69)	Inhibit
SIRT6	Glucose metabolism	Gluconeogenesis	FOXO1 deacetylation (93)	Inhibit
		Glycolysis	Corepressor of HIF-1α (94)	Inhibit
		Insulin resistance	FOXO1 deacetylation (98)	Maintain homeostasis
	Lipid metabolism	Lipogenesis	Activate AMPK pathway (102)	Inhibit
			LXRα, ChREBP, and SREBP1 deacetylation (101)	
			H3K9Ac and H3K56Ac deacetylation (103)	
		Lipolysis	FOXO1 deacetylation and insulin resistance (104)	Inhibit
		Hepatic steatosis	Attenuate USP10 deficiency-potentiated hepatic steatosis (104)	Maintain homeostasis
		Lipid accumulation	LncRNA MEG3 up-regulates SIRT6 (106)	Inhibit
		Ketogenesis	Increase Fsp27 expression in *Sirt6^-/-^* mice (107)	Enhance
SIRT7	Glucose metabolism	Gluconeogenesis	USP7 negatively regulates SIRT7 deacetylase activity by removing the K63 polyubiquitin (136)	Enhance
			Enhance the level of cellular α-KG (138)	Inhibit
			CRY1 K565/579 deacetylation (137)	Maintain homeostasis
		Glycolysis	Decrease HIF-1α expression and PGK1 K323 deacetylation (139, 140)	Inhibit
		Glucose tolerance	Decrease *ATF4* mRNA and FGF21 expression (141)	Maintain homeostasis
	Lipid metabolism	Lipogenesis	PPARγ2 K382 deacetylation (142)	Enhance
		Fatty acid uptake and triglyceride synthesis/storage	TR4/TAK1 pathway (143)	Enhance
		Lipid accumulation	Autophagy pathway (144)	Inhibit
		Lipid peroxidation	KLF15/NRF2 pathway (145)	Inhibit
		Energy consumption and thermogenesis	IMP2 K438 deacetylation (146)	Inhibit
SIRT2	Glucose metabolism	Glycolysis	iPSC reprogramming (154)	Inhibit
		Pentose phosphate pathway	G6PD K403Ac deacetylation (157)	Enhance
	Lipid metabolism	Hepatic steatosis	HNF4α deacetylation (158)	Inhibit
		Lipid deposition	Glucose intolerance and insulin resistance (160)	Inhibit
		Adipocyte differentiation	FOXO1 deacetylation (161)	Inhibit
SIRT3	Glucose metabolism	Glycolysis	GOT2 K159Ac deacetylation in malate–aspartate shuttle (179)	Inhibit
			Regulate the accumulation of HIF-1α and PKM2 dimer (181)	Maintain homeostasis
		Insulin signaling	Promoted glucose processing and PDHA1 K336Ac deacetylation (182, 183)	Maintain homeostasis
	Lipid metabolism	Lipogenesis	LCAD deacetylation or inhibition of SCD1 (186)	Inhibit
		Fatty acid β-oxidation	LCAD deacetylation (186, 187)	Enhance
			PDH activity (183)	
		Hepatic steatosis	5-hydroxytryptamine synthesis (190)	Enhance
		Lipid accumulation	LCAD deacetylation (192)	Inhibit
			SIRT3-AMPK/UCP1 pathway (193)	
			IDH2 deacetylation (195)	
		Lipid mobilization	AMPK pathway (196)	Enhance
	Amino acid metabolism	Glutamine metabolism	mTOR-PGC-1α-SIRT3 pathway (197)	Ongoing research
			TCA cycle (198)	
SIRT4	Glucose metabolism	Glycolysis	HIF-1α pathway (212)	Inhibit
		Insulin secretion	GDH activity, insulin-degrading enzymes (213)	Inhibit
	Lipid metabolism	Lipogenesis	mTORC1- SREBP1 pathway (216)	Enhance
		Fatty acid oxidation	MCD K471 deacetylation (216, 217)	Inhibit
			Repression of PPARα activity (218)	
			Expression of mitochondria and fatty acid metabolic enzymes (219)	
	Amino acid metabolism	Glutamine metabolism	mTORC1 pathway (220)	Inhibit
			Regulation of GLT-1 and GDH expression (225)	Enhance
		BCAA catabolism	Control the acylation status of enzymes (227)	Enhance
SIRT5	Glucose metabolism	Glycolysis	GAPDH K184 demalonylation (237)	Enhance
			PDHA1 K351 deglycosylation (239)	Inhibit
		Insulin secretion	PDX1 H4K16 deacetylation (240)	Maintain homeostasis
			GSTP1 demalonylation (241)	
	Lipid metabolism	Adipocyte development	Regulation of PPARγ and PRDM16 expression (243)	Enhance
		Fatty acid β-oxidation	Metabolism-related proteins demalonylation and desuccinylation (245)	Enhance
		Ketogenesis	Pyruvate and succinic acid-driven oxygen consumption (246)	Enhance
	Amino acid metabolism	Glutamine metabolism	GLS K164 desuccinylation (247)	Enhance
			PDHA1 desuccinylation (248)	Ongoing research

In the context of glucose metabolism, SIRTs are involved in in various aspects of glycolysis and insulin signaling regulation. Nuclear SIRTs specifically mediate gluconeogenesis. Among them, SIRT1 and SIRT6 regulate gluconeogenesis by downregulating FOXO1 acetylation levels. Specifically, SIRT1 enhances gluconeogenesis through FOXO1 deacetylation, while SIRT6 exerts the opposite effect. SIRT6 also controls insulin sensitivity by modulating FOXO1 activity. Furthermore, all SIRTs, except for SIRT2 and SIRT5, regulate glycolysis via HIF-1α modulation. Based on current research findings, only SIRT2 is involved in the PPP.

SIRTs exhibit higher sensitivity toward lipid metabolism than to glucose metabolism. Mitochondrial SIRTs are primarily focused on modulating lipogenesis and fatty acid oxidation, while SIRT5 and SIRT6 play significant roles in ketogenesis. Apart from SIRT2, SIRT3, and SIRT6, other SIRTs participate in regulating PPAR transcriptional activity to influence lipogenesis, lipid accumulation, and fatty acid oxidation. Moreover, SIRT3 mediates lipid metabolism by deacetylating LCAD. Additionally, the AMPK pathway is widely implicated in SIRTs -mediated lipid metabolism regulation, encompassing processes including lipogenesis, lipid mobilization and accumulation.

Interestingly, SIRT7 exhibits distinct characteristics in glucose metabolism and lipid metabolism relative to those of SIRT1 and SIRT6. Specifically, SIRT7 positively influences gluconeogenesis, while SIRT6 demonstrates an inhibitory effect on this process. Moreover, while both SIRT1 and SIRT6 stimulate lipolysis in WAT, SIRT7 promotes lipogenesis in this context (54).

Additionally, SIRT3, 4, and 5 also play key roles in regulating amino acid metabolism within mitochondria, with a primary focus on glutamine metabolism. Specifically, both SIRT3 and SIRT4 are involved in modulating glutamine metabolism through the mTOR signaling pathway. Furthermore, SIRT4 specifically regulates BCAA metabolism; for example, leucine metabolism.

**Figure 8:**
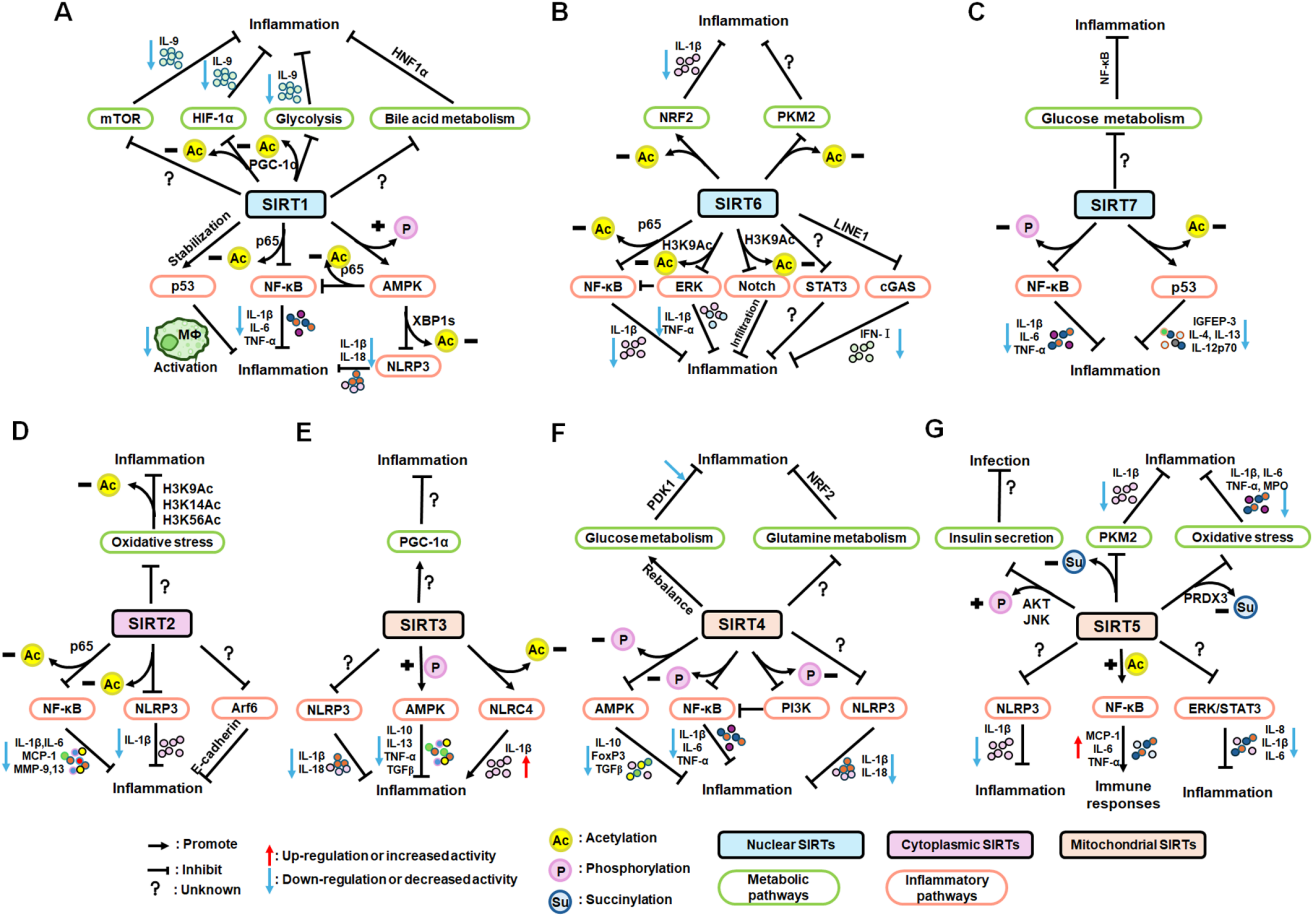
Summary of the roles of SIRT1–7 in inflammation via multiple and complex pathways, including metabolic and inflammatory pathways. **(A)** Nuclear SIRT1 regulates inflammatory processes by modulating mTOR, HIF-1α signaling and glycolysis. Furthermore, SIRT1 regulates intestinal inflammation through bile acid metabolism and HNF1α pathway. And SIRT1 regulates inflammation through the NF-κB, AMPK, p53, and NLRP3 pathways mainly through its deacetylation. **(B)** NRF2 and PKM2 are two notable pathways for inhibition of inflammatory responses by SIRT6. Through deacetylating NRF2 and PKM2, SIRT6 achieves anti-inflammatory functions. Additionally, SIRT6 blocks the NF-κB, TLR4, ERK1/2, STAT3, and cGAS signaling pathways and reduces the secretion of cytokines to play a key role in anti-inflammation. **(C)** Nuclear SIRT7 regulates anti-inflammation via glucose metabolism and NF-κB pathway. Also, SIRT7 inhibits the nuclear translocation of NF-κB p-p65 and deacetylates p53 to achieve the anti-inflammation. **(D)** Cytoplasmic SIRT2 modulates inflammation a by inhibiting oxidative stress. SIRT2 deacetylates NF-κB p65 and NLRP3 and decreases the expression of cytokines to inhibit inflammation in macrophages. Moreover, SIRT2 suppresses inflammation via inhibition of Arf6 pathway. **(E)** Mitochondrial SIRT3 regulates inflammation mainly by PGC-1α, NLRP3, AMPK and NLRC4 pathway. **(F)** For mitochondrial SIRT4, glucose and glutamine metabolism are the main metabolic pathways to inhibit inflammation. Also, SIRT4 suppresses inflammation by decreasing the phosphorylation of AMPKα, p-PI3K, p−AKT, and NF−κB p−p65. And SIRT4 inhibits the NLRP3 inflammasome to alleviate inflammation. **(G)** Mitochondrial SIRT5 has a potential role in regulating insulin secretion to suppressing inflammation. SIRT5 can modulate inflammation by regulating metabolic processes, such as PKM2, and mitochondrial oxidative stress via its desuccinylation. Additionally, SIRT5 reduces the inflammatory response via the NLRP3 inflammasome, NF-κB and ERK/STAT3 pathway.

The diagram in **Figure 8** provides a comprehensive overview of the significant impact exerted by SIRTs in modulating inflammatory responses. SIRTs can regulate inflammation through various inflammatory pathways. Simultaneously, this review also focuses on the metabolic mechanisms through which SIRTs regulate inflammation, which can also be seen in **Table 2**. For example, SIRT1 can modulate metabolic pathways, such as glycolysis, bile acid metabolism, mTOR, and HIF-1α pathways to alleviate allergic airway and intestinal inflammation, while SIRT4 can mediate inflammation by rebalancing homeostasis between glycolysis and glucose oxidation and regulating glutamine metabolism. Additionally, SIRT7 also regulate inflammation via glucose metabolism, but the special mechanisms are still unknown. Further, SIRT5 and SIRT6 affect inflammation occurrence and development through the PKM2 pathway, and both SIRT2 and SIRT5 modulate inflammation by inhibiting oxidative stress. In macrophages, SIRT3 regulates PGC-1α signaling pathway to modulate inflammation; however, the detailed mechanisms underlying SIRTs mediation of inflammatory responses through metabolic processes still need further study.

**Table 2:** Summary of the main inflammatory mechanisms regulated by SIRTs.

SIRTs	Inflammatory cells	Inflammatory mediators	Inflammatory pathways	Inflammation & Metabolism or oxidant stress
SIRT1	Macrophages (77, 80)	TNF-α (75, 76)	NF-κB pathway (77, 80)	mTOR pathway (70)
		IL-6 (79)	AMPK pathway (84, 85)	HIF-1α pathway (70)
	DC (30, 74)	IL-1β (79)	p53 pathway (86)	Glycolysis metabolism (70)
		ICAM-1 (79)	NLRP3 pathway (87)	Bile acid metabolism (73)
SIRT6	Macrophages (115, 116)	TNF-α (119)	NF-κB pathway (124)	NRF pathway (110–112)
	T cells (115)	IL-6 (121)	ERK1/2 pathway (128, 129)	
		IL-1β (122)	Notch pathway (130, 131)	
	Neutrophils (100)	ICAM-1 (79)	STAT3 pathway (132)	PKM2 pathway (91)
			cGAS pathway (133)	
SIRT7	Ongoing research	TNF-α (150)	NF-κB pathway (80, 151)	Glucose metabolism (147)
		IL-6 (150)	p53 pathway (152)	
		IL-1β (150)		
SIRT2	Macrophages (165)	TNF-α (168–170)	NF-κB pathway (171)	Oxidative stress (163, 164)
	Eosinophils (166)			
		IL-6 (168)	NLRP3 pathway (173)	
	Neutrophils (165)			
		IL-1β (168)	Arf6 pathway (174)	
	Mast cells (167)			
SIRT3	Macrophage (201)	TNF-α (204, 205)	NLRP3 pathway (206, 207)	PGC-1α pathway (200)
		IL-6 (205)	AMPK pathway (209)	
		MIP2 (205)	NLRC4 pathway (210)	
SIRT4	Treg cell (230)	TNF-α (231)	NF-κB pathway (232, 233)	Glycolysis (228)
		IL-6 (231)		
			NLRP3 pathway (235)	Glucose oxidation (228)
		MMP-9, MMP-13 (231, 234)		
SIRT5	Macrophages (250)	IL-1β (254)	NF-κB pathway (254)	Insulin secretion (249)
		IL-18 (249)	STAT3 pathway (255)	PKM2 pathway (250)
				Oxidative stress (251)

Nuclear-localized SIRTs modulate the expression of genes related to both metabolism and inflammation, indicating a greater inclination toward regulation of inflammation onset. In contrast, mitochondrial-localized SIRTs (SIRT3–5) often regulate metabolism, rather than directly influencing inflammatory processes; thus, studies investigating the roles of these specific SIRTs in inflammation regulation are limited. Exploring the inflammatory effects of mitochondrial-localized SIRTs (including SIRT7) may provide a novel direction for future research.

The impacts of the SIRTs on inflammatory responses, including the modulation of inflammatory cells, mediators, and pathways, are summarized in **Table 2**. SIRTs, particularly SIRT1, SIRT2, SIRT3, SIRT5, and SIRT6, play crucial roles in regulating macrophage activation. In certain inflammatory responses, SIRT2 also governs the functions of neutrophils, eosinophils, and mast cells, and is closely associated with their involvement in parasitic infections. Furthermore, SIRT6 influences neutrophil function by regulating glycolysis and the TCA cycle; however, eosinophil function is not influenced by other members of the SIRTs. Moreover, SIRT1 in DC has a pivotal role in regulating T cell differentiation, while SIRT4 can suppress infiltrating Tregs; however, the precise metabolic pathways through which SIRTs participate in immune cell regulation remain unknown. Nevertheless, this area holds promising research potential.

SIRT1–4, SIRT6, and SIRT7 exert anti-inflammatory effects by reducing the inflammatory responses mediated by TNF-α, IL-6, and IL-1β. Moreover, SIRT1 and SIRT6 inhibit inflammation by downregulating pro-inflammatory cytokines, such as ICAM-1. Furthermore, activation of SIRT3 modulates MIP2 production and inflammasome activation in macrophages, while SIRT2 suppresses inflammatory responses, leading to decreased MMP-9 and MMP-13 levels. In the future, further investigations are required to elucidate the precise molecular mechanisms underlying the impact of SIRTs on inflammatory factor secretion. Additionally, understanding of the role of SIRT5 in mediating inflammatory cytokine secretion warrants additional attention.

Inflammatory pathways, such as NF-κB and NLRP3 signaling, play crucial roles in inflammation development. NF-κB is a transcription factor that is indispensable for inflammatory responses and a key molecule linking chronic inflammation to cancer (257). NLRP3 acts as an intracellular sensor that detects various endogenous danger signals and environmental irritants, leading to the formation and activation of the NLRP3 inflammasome (258). Apart from SIRT3, most SIRTs exert anti-inflammatory effects by inhibiting the NF-κB pathway to modulate macrophage polarization, activation, efferocytosis, and infiltration. Furthermore, SIRT1–4 exacerbate inflammation by activating the NLRP3 pathway. Additionally, AMPK, p53 (a tumor suppressor protein), the Notch signaling pathway component, ERK, STAT3, and cGAS also contribute significantly to inflammatory progression in different disease models. Some research issues remain unresolved and will be focal points for subsequent investigations. For example, whether SIRT3 influences the NF-κB pathway remains undetermined and if SIRT5–7 participate in modulating inflammation through the NLRP3 pathway requires further exploration. In addition to extensive study of the NF-κB and NLRP3 pathways, investigating the novel precise mechanisms underlying SIRTs -mediated inflammatory pathway regulation across diverse disease contexts represents a promising avenue for future exploration.

SIRTs also exhibit compensatory and synergistic effects in specific inflammation (259). Interestingly, SIRT1 and SIRT2 have important roles in restraining premature activation of inflammatory genes during macrophage differentiation (260). Moreover, SIRT1 and SIRT6 impede TNF-α-induced inflammation in vascular adventitial fibroblasts through the ROS and AKT pathways by inhibiting MCP-1 and IL-6 expression (261). Furthermore, simultaneous deletion of both SIRT2 and SIRT3 influences the metabolism and inflammatory responses of macrophages, while providing protection against endotoxemia; however, a single deficiency in either SIRT2 or SIRT3 has minimal or no impact on antimicrobial innate immune responses (259). Additionally, individual deficiencies in either SIRT3 or SIRT5 do not affect host defenses; nevertheless, mice deficient in both SIRT3 and SIRT5 display enhanced inflammatory and bactericidal responses to *Listeria monocytogenes* infection, including alterations to the inflammatory profiles of macrophages, as well as increased killing activity (262).

## 6 The development of compounds targeting SIRTs in inflammation

Drugs targeting SIRTs are currently in the research stage, with only a few having entered the animal models and clinical practice (**Table 3**). Some compounds targeting nuclear SIRT1, SIRT6 and cytoplasmic SIRT2 are now being or have already been tested in animal models. However, current research on nuclear SIRT7, and mitochondrial SIRT3–SIRT5 is still in the basic experimental stage.

**Table 3:** Summary of the structure and function of some compounds targeting SIRTs with clinical application prospects.

SIRTs	Compounds		Structure	Function
SIRT1	Activators	SRT2104(**1**)	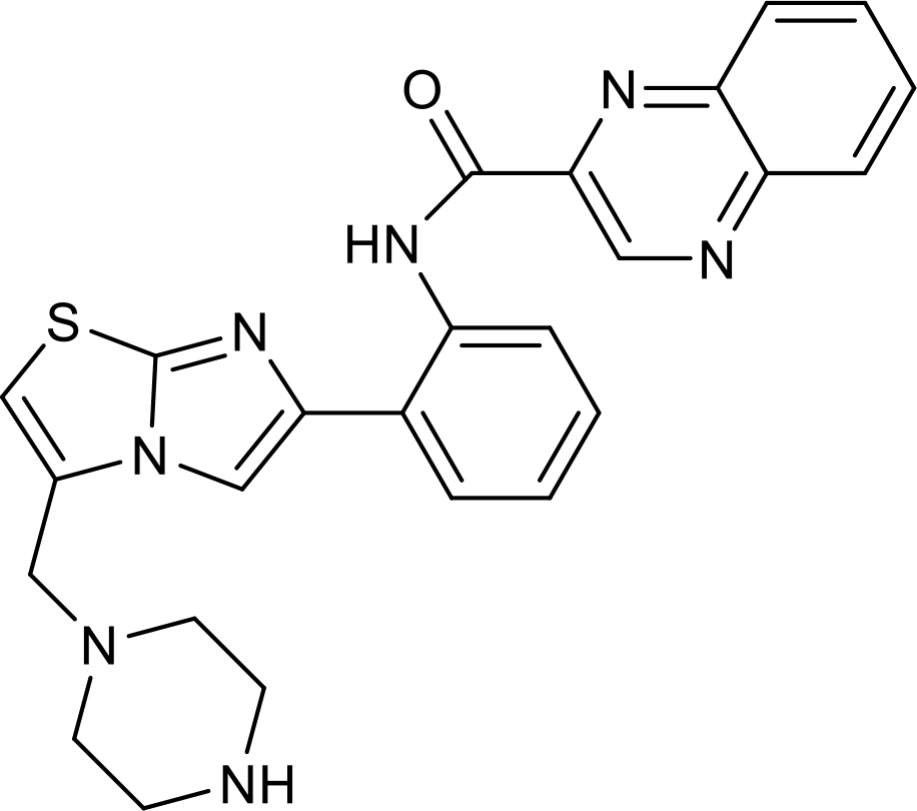	Inhibit systemic inflammatory responses in human (263)
		SRT1720(**2**)	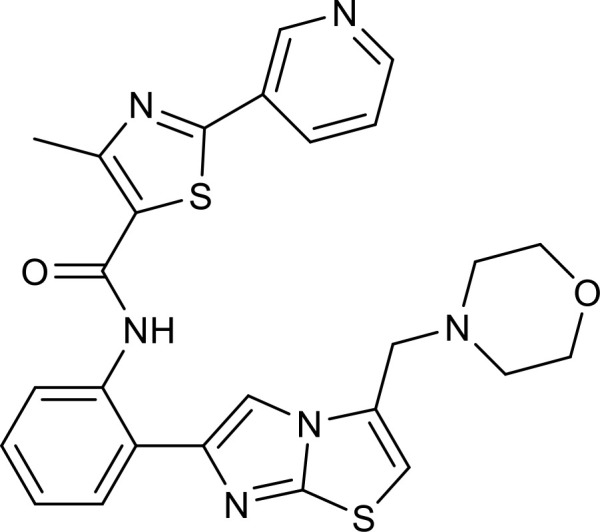	Reduce intrahepatic cholestasis produced by inflammation (264)
		SRT2183(**3**)	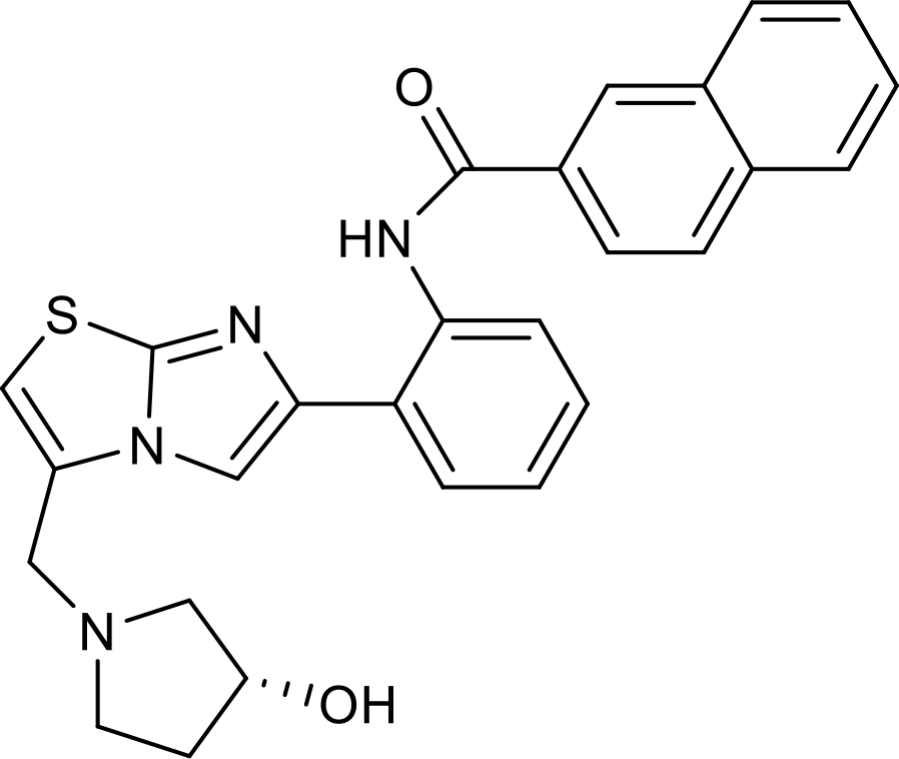	Currently under investigation
		SRT1460**(4)**	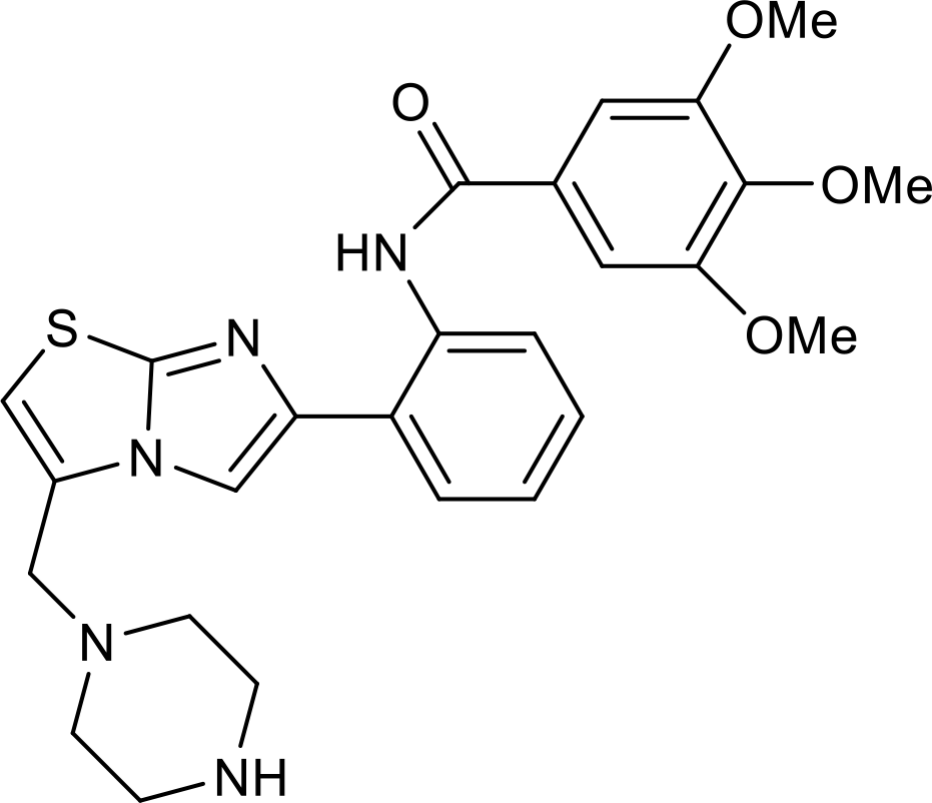	Currently under investigation
SIRT6	Activators	MDL-800(**5**)	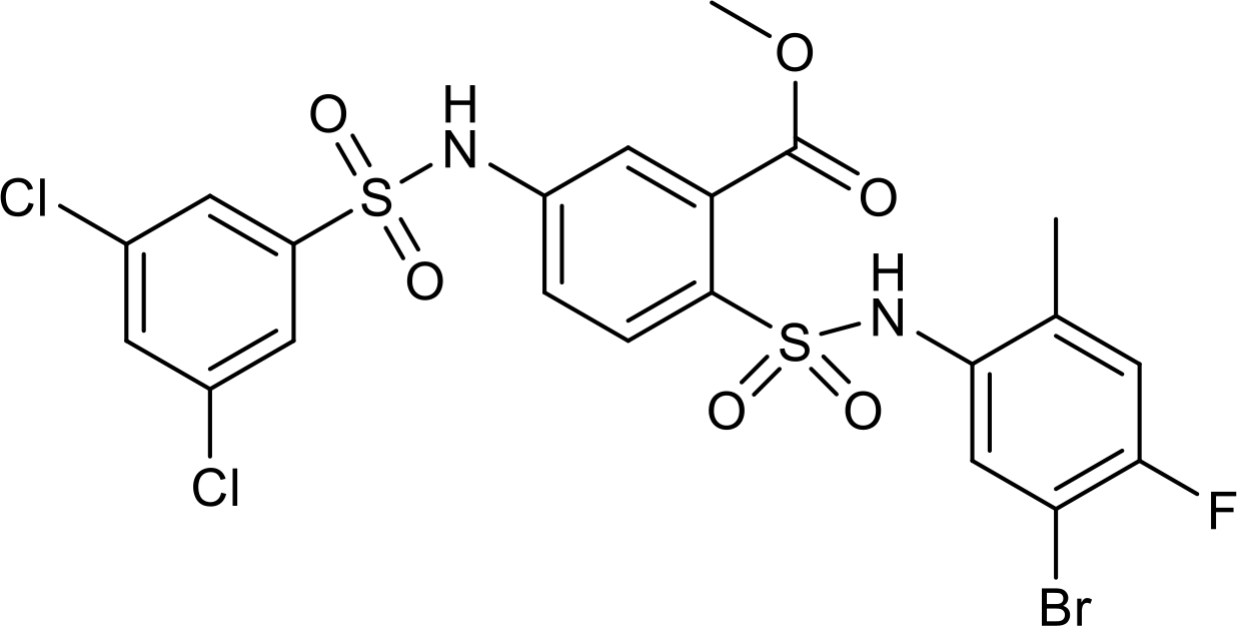	Suppress inflammation (121)
		MDL801(**6**)	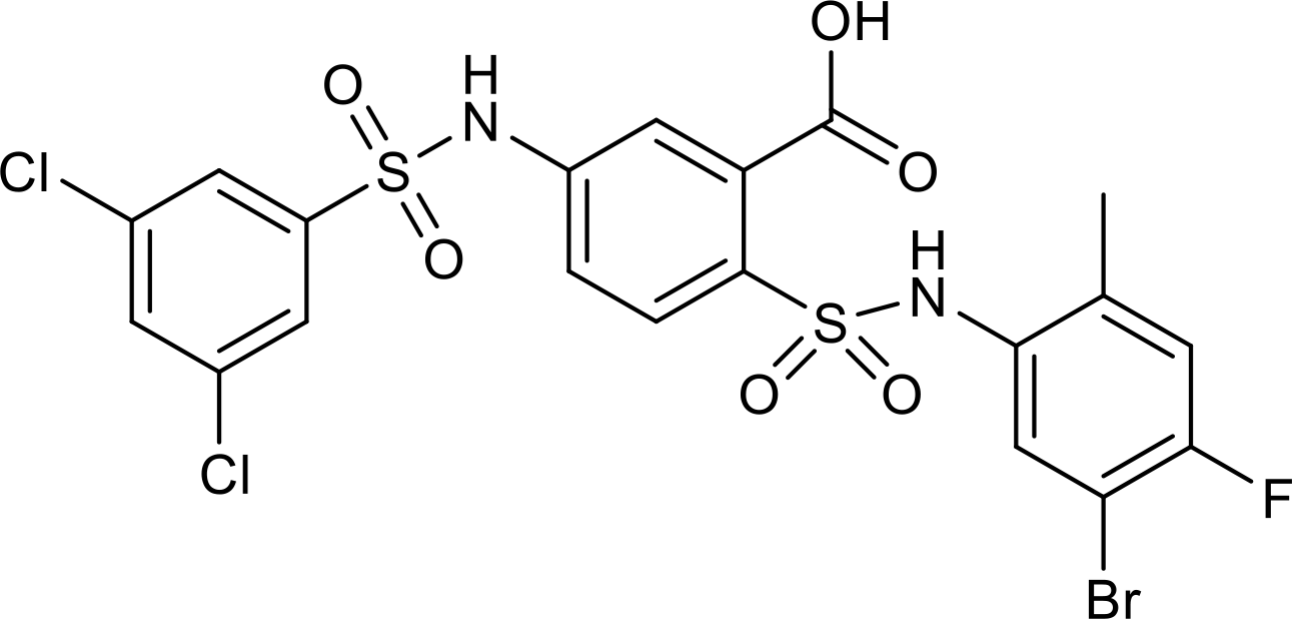	Ameliorate inflammatory bone loss (266)
		MDL-811(**7**)	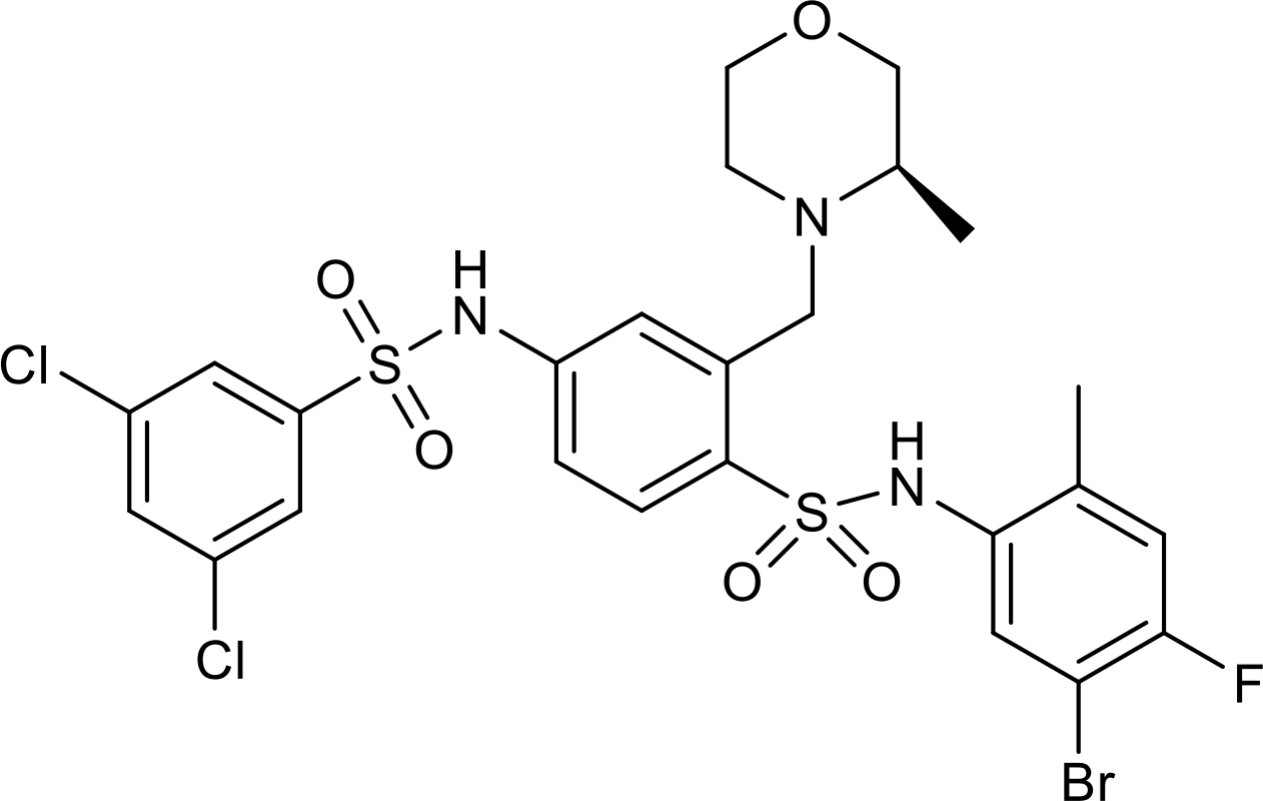	Inhibit inflammatory response in mice microglia (267)
		Chrysophanol(**8**)	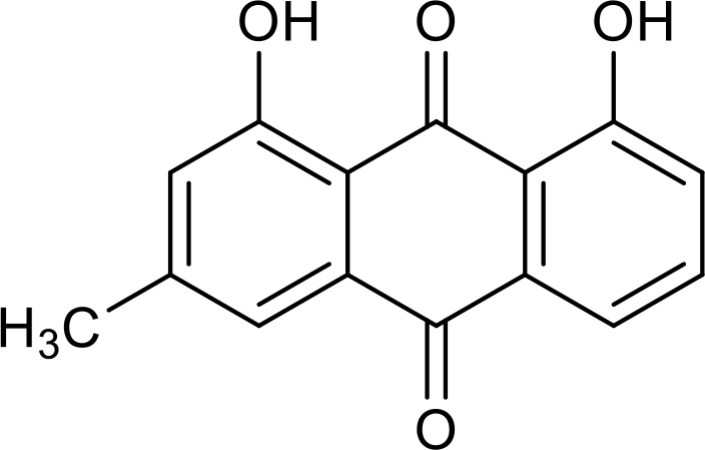	Impede osteoarthritis (268)
		Orientin (**9**)	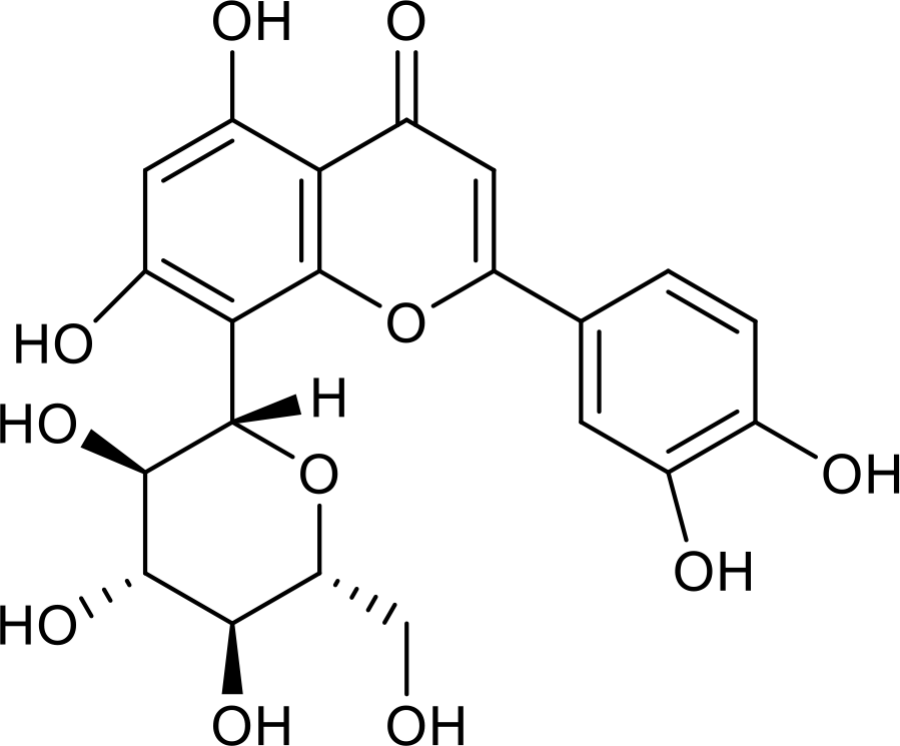	Attenuates IL-1β-induced inflammation (113)
SIRT7	Currently under investigation			
SIRT2	Inhibitors	AK-7(**10**)	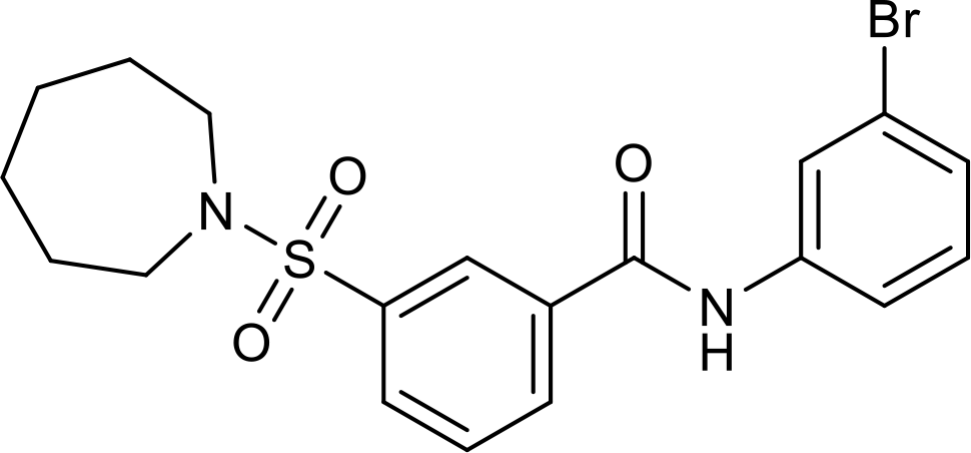	Decreases TNF-α and IFN-γ levels (269)
		AGK2**(11)**	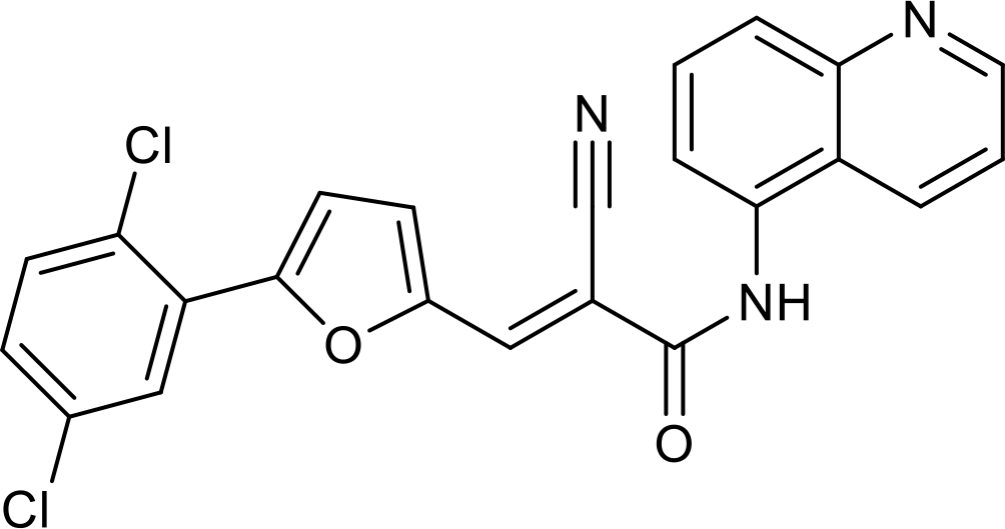	Ameliorate mast cell-mediated allergic airway inflammation (167)
SIRT3	Currently under investigation			
SIRT4	Currently under investigation			
SIRT5	Currently under investigation			

A selective SIRT1 activator, SRT2104(**1**), reduces endotoxin-induced cytokine release and coagulation activation and inhibits systemic inflammatory responses in human (263). SRT1720(**2**), a SIRT1 activator, reduces intrahepatic cholestasis caused by inflammation and bile acid accumulation in mice models of cholestasis (264). However, SRT1720 has not yet officially entered large-scale clinical trials. The results vary depending on the method. Pacholec et al. reported that small compounds [e.g., SRT1720(**2**), SRT2183(**3**), SRT1460(**4**)] used to modulate SIRTs have many off-target effects by using biochemical experiments with native substrates, such as a p53-derived peptide substrate without a fluorophore, the purified native full-length protein substrates p53 as well as acetyl-CoA synthetase1 (265). SRT2104 was used in the clinical trials, which encouraged the further development of more SIRTs-targeting compounds. However, there are conflicting results that could arise from different approaches to modulate SIRTs to study their effects.

SIRT6 could be a useful target for therapy. Many reports suggest that SIRT6 can be activated by small molecules to play its role in inflammation. MDL-800(**5**), the SIRT6 activator, suppresses inflammation via the NF-κB pathway and downregulates the expression of inflammatory factors, which promotes angiogenesis to accelerate cutaneous wound healing in mice (121). SIRT6 activation by MDL801(**6**) alleviates inflammatory bone loss in ligation-induced periodontitis and downregulates the expression of pro-inflammatory cytokines in mice (266). SIRT6 activated by MDL-811(**7**) directly promotes deacetylation of EZH2 and inhibits inflammatory response in LPS-stimulated RAW264.7 macrophages and mice primary microglia (267). Chrysophanol(**8**) could impede the NF-κB pathway of IL-1β-induced chondrocytes of osteoarthritis mice by activating SIRT6 (268). Orientin(**9**), a natural flavonoid compound, attenuates IL-1β-induced inflammation and extracellular matrix degradation through activating the NRF2/HO-1 and the SIRT6 signaling pathways (113). However, research on these compounds is still in the preclinical stage.

The current research focuses on the development and implementation of SIRT2 inhibitors. AK-7(**10**) significantly reduces apoptosis, TNF-α and IFN-γ levels in cigarette smoke-induced mice through regulation the *Nrf2* (269). AGK2(**11**), ameliorates mast cell-mediated allergic airway inflammation and fibrosis by inhibiting FcϵRI/TGF-β signaling pathway (167). To date, AK-7, AGK2, and other SIRT2-targeting compounds have previously demonstrated potential therapeutic benefits on inflammation in basic investigations. However, additional research is required to confirm the safety and effectiveness of these substances before they can be advanced to clinical trials.

Although the potential therapeutic role of SIRT7 and SIRT3–5 is widely recognized, there is currently a lack of highly potent and selective SIRTs inhibitors or activators that can be used in clinical trials. Traba et al. discovered that nutrient levels regulate the NLRP3 inflammasome, in part through SIRT3 in a clinical study of 19 healthy volunteers (270). These findings expand the understanding of the crucial role of SIRTs in metabolism and inflammation while providing a preclinical rationale for inhibiting abnormal metabolism and inflammation by modulating the expression and activity of SIRTs in patients.

With an enhanced understanding of the biological functions of SIRTs, researchers may identify novel compounds that modulate the activity of SIRTs to regulate inflammatory processes. It is anticipated that an increasing number of targeting SIRTs compounds will advance to preclinical studies and clinical trials in the future.

## 7 Conclusions and perspectives

In this review, we summarize the crucial roles of SIRTs, as epigenetic regulators, play crucial roles in modulating cell metabolism and inflammation as epigenetic regulators. SIRTs exert their anti-inflammatory effects through diverse metabolic pathways. In general, SIRTs regulate glucose, lipid, and amino acid metabolism to finely tune inflammatory responses. The involvement of SIRTs in inflammation is primarily associated with epigenetic modifications, which are closely linked to intracellular NAD^+^ levels. These multifunctional protein PTMs contribute extensively to metabolic and inflammatory signaling pathways. Although numerous acetylated proteins regulated by SIRTs have been reported to date, other lysine acylation modifications, such as lactylation, glutarylation, 2-hydroxyisobutyrylation, and benzoylation, have received limited attention, possibly due to their lower prevalence within inflammatory cells. Henceforth, it will be crucial to understand the precise molecular basis of these novel PTMs, to provide valuable insights that can inform targeting of SIRTs -mediated metabolism and inflammation. Moreover, future research should focus on identifying novel substrates, or potential metabolic pathways, regulated by SIRTs that can contribute to precise and improved treatment strategies for specific diseases, while providing valuable insights to inform the future development of precision medicines.

For example, nuclear receptors, such as HNF1α, are regulated by PTMs (acetylation, methylation, and phosphorylation), which significantly impact their stability and transcriptional activity (271, 272). SIRT1 and HNF-1α interact physically, and deletion of SIRT1 in the intestinal epithelium reduces HNF-1α/farnesoid X receptor (FXR) signaling and attenuates ileal bile acid absorption (73, 273). Recent research has highlighted the bile acid biosynthetic pathway (e.g., 3-sucCA) as an additional metabolic route implicated in chronic inflammation (72), underscoring the importance of exploring new metabolic pathways to comprehension of inflammation pathogenesis. Hence, research efforts should be directed towards investigating how SIRT1 regulates biosynthesis of bile acids through modulating various acylation levels in its potential substrates, including HNF1α, which will offer potential novel and valuable targets for treatment of chronic inflammation.

The details of inflammation regulation by SIRTs are becoming increasingly clear; however, many unanswered questions remain. Further investigation is needed to determine whether regulating the activity of SIRTs alone can effectively reverse inflammation. The roles of SIRTs in inflammation regulation are intricately related, and certain SIRTs, such as SIRT7, exhibit varying roles in different diseases (30).

In conclusion, due to their crucial involvement in inflammation regulation, SIRTs have emerged as potential therapeutic targets for combatting acute or chronic inflammatory diseases, based on current research. However, extensive work is still required to pinpoint the exact molecular mechanisms governing the functions of SIRTs under various disease conditions. Small molecule activators or inhibitors targeting specific SIRTs -associated substrates or metabolic pathways are expected to enable precise treatment for specific diseases. In the future, researchers should focus on determining whether promising compounds targeting SIRTs affect anti-inflammatory activity by participating in metabolic pathways in inflammatory cells, which represent common and promising targets in both metabolic diseases and inflammatory responses.

While the therapeutic potential of SIRTs in various diseases has been widely acknowledged, the clinical application of compounds targeting SIRTs remains in the development phase. Most research has focused on animal models. Clinical trials, however, are not lacking in significant challenges. For instance, the expression and activity of SIRTs may differ between patients. Furthermore, hurdles in the investigation of SIRTs modulators include ensuring modulator specificity and minimizing potential side effects. Future paths for research might involve investigating the therapeutic potential of SIRTs in various disorders, as well as developing tissue-specific SIRTs inhibitors or activators.
